# Novel insights into TCR-T cell therapy in solid neoplasms: optimizing adoptive immunotherapy

**DOI:** 10.1186/s40164-024-00504-8

**Published:** 2024-04-03

**Authors:** Weihuan Shao, Yiran Yao, Ludi Yang, Xiaoran Li, Tongxin Ge, Yue Zheng, Qiuyi Zhu, Shengfang Ge, Xiang Gu, Renbing Jia, Xin Song, Ai Zhuang

**Affiliations:** 1 Department of Ophthalmology, Ninth People’s Hospital, Shanghai JiaoTong University School of Medicine, 639 Zhi Zao Ju Road, Shanghai Ninth People’s Hospital, Shanghai, 200011 People’s Republic of China; 2 Shanghai Key Laboratory of Orbital Diseases and Ocular Oncology, Shanghai, 200011 People’s Republic of China

**Keywords:** T cell receptor, TCR-T cell, Immunotherapy, Solid tumor, Tumor antigen, Clinical application

## Abstract

Adoptive immunotherapy in the T cell landscape exhibits efficacy in cancer treatment. Over the past few decades, genetically modified T cells, particularly chimeric antigen receptor T cells, have enabled remarkable strides in the treatment of hematological malignancies. Besides, extensive exploration of multiple antigens for the treatment of solid tumors has led to clinical interest in the potential of T cells expressing the engineered T cell receptor (TCR). TCR-T cells possess the capacity to recognize intracellular antigen families and maintain the intrinsic properties of TCRs in terms of affinity to target epitopes and signal transduction. Recent research has provided critical insight into their capability and therapeutic targets for multiple refractory solid tumors, but also exposes some challenges for durable efficacy. In this review, we describe the screening and identification of available tumor antigens, and the acquisition and optimization of TCRs for TCR-T cell therapy. Furthermore, we summarize the complete flow from  laboratory to clinical applications of TCR-T cells. Last, we emerge future prospects for improving therapeutic efficacy in cancer world with combination therapies or TCR-T derived products. In conclusion, this review depicts our current understanding of TCR-T cell therapy in solid neoplasms, and provides new perspectives for expanding its clinical applications and improving therapeutic efficacy.

## Introduction

Significant advances have been made in cancer immunotherapy, and the innate immune system has a vital role against tumor progression in the tumor microenvironment (TME), particularly in solid tumors [1]. Adoptive cell transfer (ACT) therapy, in combination with immune checkpoint inhibitors (ICIs), can induce tumor regression [2–5]. ACT represents a pioneering immunotherapy distinguished by its wide-ranging applicability, which has contributed to its rapid progress and therapeutic breakthroughs. ACT has evolved over several generations from autologous tumor-infiltrating lymphocyte (TIL) therapy [6, 7] to antigen-specific endogenous T cell therapy, culminating in chimeric antigen receptor (CAR) and TCR-T cell therapies. And the manufacturing process for ACT has developed from simple targeting of T cell populations for expansion from the TME, into now the use of genetic engineering in both CAR-T and TCR-T cells. This involves both modification of autologous or donor T cells and expansion [8] to obtain functionally engineered T cell populations aimed at specific target peptides.

CAR-T cells directly recognize extracelluar membrane antigens, eliminating restrictions related to major histocompatibility complex (MHC) extraction and delivery [9]. Mature applications of CAR-T cell therapy have been developed for a variety of hematologic malignancies, leading to remarkable progress in the treatment of B cell leukemia and B cell lymphoma [10, 11]. As of 2023, eight applications had been approved by the Food and Drug Administration, starting with Tisagenlecleucel, the first anti-CD19 CAR-T product to be approved. However, CAR-T cells can only recognize cell surface antigens, and there are difficulties in the treatment of solid tumors owing to the heterogeneity of cellular antigens, limitations in tumor-associated antigen (TAA) library to target [12, 13], and challenges of infiltration and T-cell depletion in TME [14–16]. It is hoped that with fourth- and next-generation CAR-T cell therapies, improved safety and a controlled therapeutic window will be achieved by co-expression of cytokines or other co-receptors [17, 18]. Also, in some clinical trials, CAR-T cells targeting the oncofetal antigen claudin-6 [19] and claudin 18 [20] for the treatment of solid tumors have been shown to be feasible.

TCR-T cells function via exogenously specific TCR to achieve CD8 cytotoxic T lymphocyte (CTL) lytic activity. TCR-T cells strictly recognize peptide epitopes presented by MHC class I molecules. Cytoplasmic proteasome breakdown and delivery of MHC molecules by means of biofilm fluidity make it possible for neoplasm cells to display diverse antigens. The innate cytosolic pathway creates more antigen targets for TCRs in whole-cell fractions to recognize and affinite. Sharable antigens for solid tumors remain to be found, and the therapeutic efficacy of TCR-T has been clinically validated. More critically, a range of tumor neoantigens has been identified, and some clinical trials have demonstrated the efficacy of TCR-T cell therapy against metastatic solid tumors [21–23]. In this review, first, we introduce the tumor-antigen libraries and the process of TCR acquisition and optimization. Second, we summarize the construction and application of TCR-T cells, including computer simulations and the complete flow from laboratory to clinic. We also discuss the methods for enhancing TCR-T affinity instead of cross-reactivity. Third, the constraints and future prospects for improving the efficacy of TCR-T cell therapy in cancer treatment are considered. In conclusion, this review elucidates current clinical applications of TCR-T cell therapy on solid neoplasms.

## TCR-T design and manufacturing

### Principles

Αβ TCR multimers activate the TCR signaling pathway by recognizing and binding extracellularly to peptide-MHC (p-MHC) complexes. These consist of a predominant heterodimeric duplex, usually paired by one α chain and one β chain, both of which have a transmembrane region and an antigen-binding region. The TCR chain is anchored to the T cell membrane by the constant region, and each chain can be reconfigured by rearrangement of variable regions [24]. Such rearrangements, which involve random and exponential differences, represent a good implementation of the inherent polymorphism and reserve of TCRs and can theoretically handle any epitope sequence. Rearranged TCRs then undergo physiological maturation of affinity through thymic selection [25]. The TCR recognizes and binds the p-MHC complex through interactions of its two variable regions [26]. TCRs do not function independently; activation of T cells depends on the cooperation of the TCR heterodimer and CD3 six-chain multimers, which comprise three dimers: one CD3 γε heterodimer, one CD3 δε heterodimer, and one CD3 ζζ homodimer [27, 28].

**Fig. 1:**
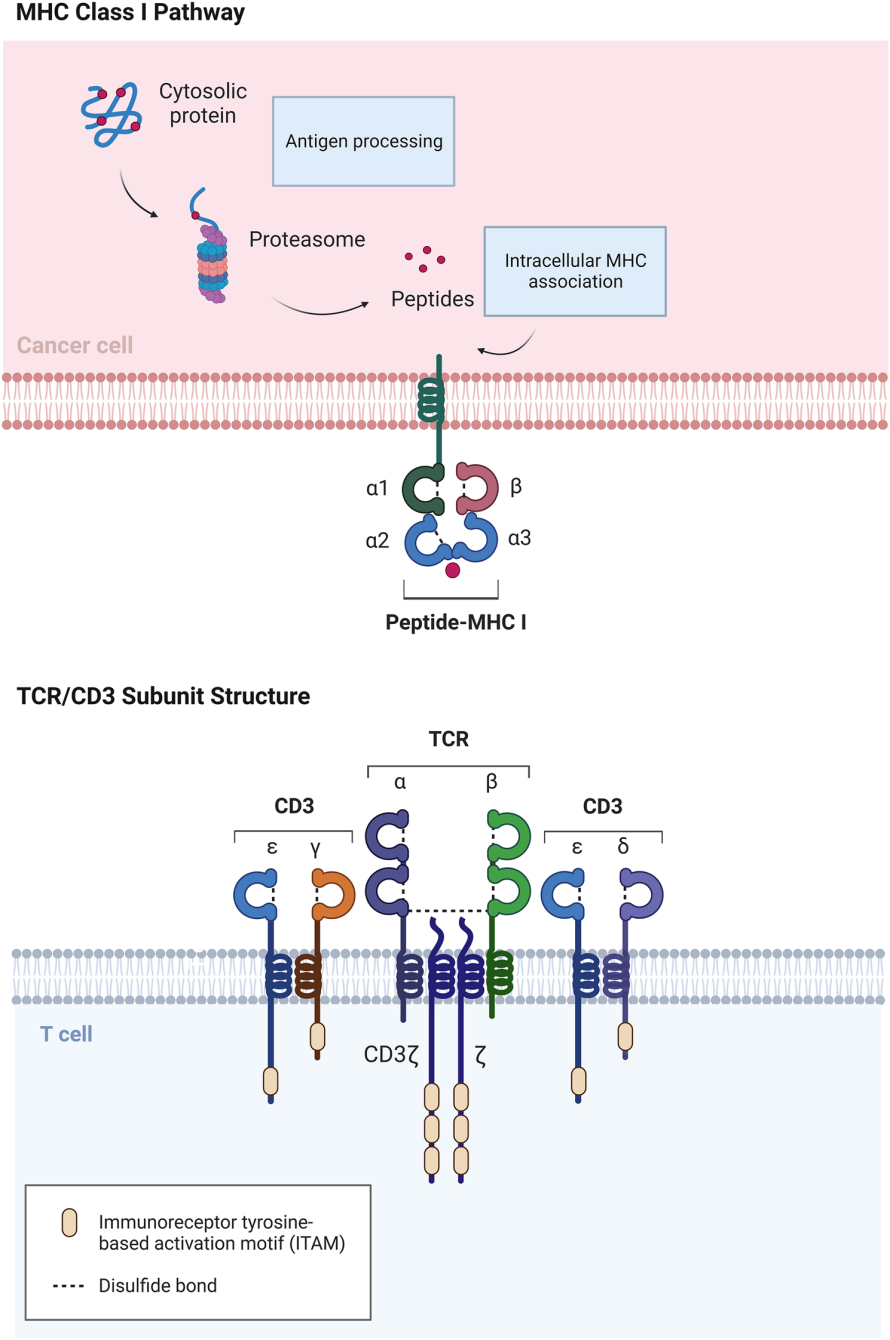
Schematic diagram of TCR/CD3 and peptide-MHC structure. Cancer cells process and deliver intracellular antigenic peptides via MHC class I molecules on the cell membrane. The TCR complex is essentially an α, β-double-stranded heterodimer, recognizing the peptide-MHC complex with the CD3 γε heterodimer, CD3 δε heterodimer and CD3 ζζ homodimer cooperatively

Artificially engineered TCRs use similar mechanisms to those of native TCRs to recognize specific MHC complexes with the HLA isotype (Fig. 1). The killing activity of T cells is directly related to the affinity of TCRs. T cell editing can be used to achieve recognition and effects on tumor cells beyond those possible in the physiological state. This requires the expression of specific relatively high-affinity TCRs; the natural CD complexes oscillating on the cell membrane are used to build functional receptors, which are provided in part by the T cells themselves. This means that the process can be physiologically activated by the T cells; however, co-stimulatory signals are essential, including CD28 and CD137 (4-1BB) on the surface of T cells. The production of equipped TCR-T cells, the search for and exploitation of novel specific TCR-T cells, manufacturing of personalized tumor neoantigens, screening of TCR genes, and localization and isolation can be progressively simplified and made more rapid using genomics techniques. Moreover, next-generation sequencing (NGS) and single-cell multi-omics sequencing technologies for screening and identification of target proteins can be used to comprehensively determine the tumor specificity of TCR sequences and structures and predict recognizable epitopes.

### Recognition, characterization, and acquisition of TCR-T cells and p-MHC

#### Screening and identification of antigens

In order for TCR-T cells to achieve specific tumor cell killing, the antigen used as a target for TCR recognition should possess the following qualities:

1. expression solely or predominantly in tumor cell populations;
2. correlation with key tumor events;
3. known targets and coding sequence;
4. immunogenicity that can trigger a T cell response capacity;
5. no cross-reactivity with autoantigens;
6. non-rapid induction of T cell depletion.

Existing immunotherapies for solid tumors can be divided into two broad categories based on the antigens involved: TAAs and tumor-specific antigens (TSAs), also known as neoantigens [29, 30]. Clinical trials of TCR-T cell therapy that are currently underway or have been initially completed for patients with solid tumors also focus on these two antigen categories. The main known clinical trials and their outcomes are listed in Table 1. In addition, HA-1-specific targeted T cells have demonstrated favorable safety profiles in some clinical tests of ACT [31, 32]. However, the requirement for donors and ligands to have compatible single nucleotide polymorphisms (SNPs) makes it challenging to find suitable matches for recipients [33].

**Table 1:** Current major target antigens and clinical trials of TCR-T cell therapy

Antigen type	Target antigen	HLA	Cancer type	Clinical trial	Phase	Objective response rate (ORR) (%)	Clinical response	References
TDA	MART-1	HLA-A*0201	Melanoma	n.s	n.s	2/17 (12)	2 PR	[34]
	MART-1	HLA-A*02:01	Melanoma	NCT00509288	2	6/20 (30)	6PR	[35]
	MART-1	HLA-A*02:01	Melanoma	NCT00910650	2	0/13 (0)	0	[36]
	MART-1	HLA-A*02:01	Melanoma	NCT02654821	1/2a	2/12 (16.7)	2PR	[37]
	gp100	HLA-A*02:01	Melanoma	NCT00509496	2	3/16 (16)	1CR, 2PR	[35]
	CEA	HLA-A*02:01	Colorectal cancer	NCT00923806	1	1/3 (33)	1PR	[90]
CGA	NY-ESO-1	HLA-A*02:01	Melanoma; synovial sarcoma	NCT00670748	1	5/11 (45) 4/6 (67)	2CR, 3PR; 4PR	[40]
	NY-ESO-1	HLA-A*02:01	Melanoma; synovial sarcoma	NCT00670748	2	11/20 (55); 11/18 (61)	4CR, 7PR; 1CR, 10PR	[41]
	NY-ESO-1	HLA-A*02:01	Melanoma; synovial sarcoma; liposarcoma; osteosarcoma; MPNST	NCT02070406; NCT01697527	1	2/10 (20)	2PR	[42]
	NY-ESO-1	HLA-A*02:01;HLA-A*02:06	Synovial sarcoma	NCT01343043	1/2	6/12 (50)	1CR, 5PR	[43]
	NY-ESO-1	HLA-A*02:01;HLA-A*02:06	Synovial sarcoma	NCT01343043	1/2	9/30 (30)	9PR	[44]
	NY-ESO-1	HLA-A*02:01;HLA-A*02:06	Synovial sarcoma	JMA-IIA00346	1	1/3 (33)	1CR	[45]
	NY-ESO-1 (CRISPR/Cas9)	HLA-A*02:01	Metastatic sarcoma; Myeloma	NCT03399448	1	0/3	0	[46]
	NY-ESO-1	HLA-A*02:01	Myeloma	NCT01352286	1/2	11/25 (44)	1SCR,1CR, 8VGPR, 1PR	[47]
	MAGE-A3	HLA-A*02:01	Melanoma; synovial sarcoma; esophageal cancer	NCT01273181	1/2	5/9 (56)	1CR,4PR	[52]
	MAGE-A3	HLA-A*01	Melanoma	NCT01350401	1	0/1	0	[51]
	MAGE-A3	HLA-DPB1*0401	Metastatic solid tumors	NCT02111850	1	4/17 (23.5)	1CR,3PR	[53]
	MAGE-A4	HLA-A*24:02	Esophageal cancer	UMNI000002395	1	0	/	[49]
	MAGE-A4	HLA-A*02	Relapsed/refractory metastatic solid tumors (9 types)	NCT03132922	1	9/38 (24)	9PR	[50]
	MAGE-10	HLA-A*02:01 OR HLA-A*02:06	Nsclc	NCT02592577	1	1/11 (9)	1PR	[48]
	PRAME	HLA-A*02:01	Cutaneous melanoma, uveal melanoma, endometrial carcinoma, synovial sarcoma, and ovarian cancer	NCT03686124	1	8/12 (64)	8PR	[61]
Viral antigen	HPV16-E6	HLA-A*02:01	Epithelial cancer	NCT02280811	1/2	2/12 (17)	2PR	[94]
	HPV16-E7	HLA-A*02:01	Epithelial cancer	NCT02858310	1	6/12 (50)	6PR	[95]
	HBV	HLA-A*02:01;HLA-Cw0801	HBV-	NCT03899415	1	1/8 (12.5)	1PR	[96]
	MCPyV	HLA-A*02:01	Merkel cell carcinoma	NCT03412877	1	1/5 (20)	n.s	[97]
Neoantigen	TP53	HLA-A*02:01	Metastatic breast cancer	NCT03412877	1	1/1 (100)	1PR	[21]
	KRAS G12D	HLA-A*08:02	Metastatic pancreatic cancer	IND 27501	1	1/1 (100)	1PR	[22]
	mutation-associated neoantigens (CRISPR/Cas9)	multiple HLA class I	Metastatic solid tumors	NCT03970382	1	0/16	0	[23]

*MPNST* malignant peripheral nerve sheath tumor, *NSCLC* non small cell lung cancer, *PR* partial response, *CR* complete response, *SCR* strictly complete response, *VGPR* very good partial response

Our current knowledge on target antigens for TCR-T cell therapy is summarized in Table 2.

**Table 2:** Classification and description of target antigens in TCR-T cell therapy

Categories		Shared antigens and genes in solid tumors	Advantages	Disadvantages
TAAs -Endogenous wild-type proteins	Tissue differentiation antigens	• MART-1/Melan-A [34–37] • gp100 [35] TYRP1[38] • mesothelin [39]	Antigen ubiquity: widely shared between patients; widely shared under tumor heterogeneity Easier to recognize, isolate and validate Relatively mature, have undergone clinical tests	Prone to immune evasion under affinity limitation Relatively poor effect, easily tolerated Autosomal cross-reactions have been widely reported, causing multiple injuries and even death
	Cancer germline antigens	• NY-ESO-1 [40–47] • MAGE-A [48-53] • BAGE [54] • SAGE [55] • HAGE [56] [57] • SSX [58] • LAGE [59] • SCP1 [60] • PRAME[61]		
TSAs (neoantigens) -Somatic mutant proteins	Neoantigens from genomic mutations: SNVs; Indels; Fusion genes; Chromosomal structural abnormalities	SNVs • *TP53 *[21, 62, 63] • *KRAS *[22, 64–67] • *IDH1* [68] • *JAK2* [69] • *BRAF* [70] • *CDK4* [71] • *CDK12* [72] • *NRAS* [65] • *CTNNB1* [73] • *GAS7* [74] Indels • *NPM1* [75] • *CALR* [76] • *TGFBR2* [77] Fusion genes • *BRD4-NUT* [78] • *NTRK1/2/3* [79–81] • *NRG1* [82]	No expression in normal somatic cells No T cell thymocyte selection and central immune tolerance Individuation, more in line with the heterogeneity of patients with tumors, new therapeutic potential	Need to predict and characterize, more cost of time and resources Need to find commonality Immature and difficult to validate Unknown risk of cross-reactivity
	Viral neoantigens (viral open reading frames)	• HPV-16 E7 • HPV-16 E6		
	Neoantigens of transcriptomic variants	• COL6A3-FLNV [83]		
	Neoantigens of proteomic variation/ abnormal antigenic peptide presentation	• LUAD [84]		
MiHAs	A large antigen library distinct from MHC presentation		Available applications in hematologic malignancies	Strict requirement of individual matching

*TAA* tumor-associated antigen, *TSA* tumor-specific antigen, *MiHA* minor histocompatibility antigen, *SNV* single nucleotide variation

##### (1) TAAs

TAAs exhibit limited expression levels in a restricted number of cell types in normal tissues but are upregulated in tumor tissues owing to abnormal gene expression. TAAs arise from epitopes of endogenous wild-type proteins from specific populations and can be shared between different patients and tumor types, making them potentially suitable for universal immunotherapies [85, 86]. However, the recognition of these antigens by existing specific TCRs is often of low affinity owing to negative selection and tolerance processes against self-antigens in early development, leading to suboptimal clinical trial outcomes [87]. Furthermore, the engineering of high-affinity TCRs targeting TAAs may increase the risk of self-cross-reactivity to normal cells with low antigen levels. Reported severe autoimmune reactions include colitis, kidney damage, severe hepatitis, respiratory failure, and treatment-related fatalities [88].

###### Tissue differentiation antigens (TDAs)

TDAs are present in specific stages of cell differentiation but may be shared with a small number of antigens in normal cells. Melanocyte differentiation antigens currently commonly used for human TCR target include MART-1/Melan-A [34–37], gp100 [35], and tyrosinase [89]. A number of clinical trials targeting MART-1 demonstrated some clinical efficacy, but some dose-dependent toxicities were documented, illustrating the potential risk of TDA expression of normal tissues. Besides, carcinoembryonic antigen (CEA) was targeted in an early clinical study of TCR-T to three patients with metastatic colorectal cancer. All patients presented with decreased serum CEA levels and one-third example of objective regression. However, it should be noted that all patients reported the occurrence of a severe transient inflammatory colitis, and the limitations of using CEA as a target for cancer immunotherapy were similarly described [90].

###### Cancer germline antigens (CGAs)

CGAs, also called “cancer testis antigens”, are normally restricted to germ cells. CGAs have been targeted in the majority of the TCR-T cell clinical trials, with favorable objective remission outcomes. The New York Esophageal Squamous Cell Carcinoma Antigen 1 (NY-ESO-1) targeted product has achieved favorable results in clinical trials, particularly in cutaneous melanoma and synovial sarcoma [40–47]. However, it has been reported to have limited expression in metastatic cancers. Owing to the effects of tumor heterogeneity on NY-ESO-1, maintenance of long-term stability for individuals needs to be further explored [91]. Some studies have shown that preferentially expressed antigen of melanoma (PRAME), another germinal tissue-specific antigen, participates in the proliferation and survival of cancer cells in a variety of malignancies, including melanoma, sarcoma, lung cancer, head and neck cancer, and kidney cancer, as well as being expressed in healthy tissues such as gonads, adrenal glands, bone marrow, and brain [92, 93]. Recently, one clinical trial of TCR-T cell targeting PRAME has yielded a good clinical objective response rate in the trial cohort (NCT03686124) [61]. Melanoma antigens (MAGE), especially MAGE-A, have been widely used in the treatment of various solid tumors. However, clinical trials of MAGE-A10 have to date only yet reported results regarding usability in non-small-cell lung cancer [48], partly owing to the high degree of overlap between MAGE-A10 and MAGE-A4 expression. Clinical trials of MAGE-A4 have shown it to be relatively effective against synovial sarcomas [49, 50]. However, the trial of engineered T cells on MAGE-A3 has reported fatalities [51], which have been confirmed to be caused by cross-reaction with the MAGE-A12 protein in the brain. Neurotoxicity and cardiac toxicity were reported in another clinical trial [52], with two treatment-related deaths, which preliminarily demonstrated cross-reaction with actin of cardiac myocytes. After that, one clinical trial of engineered MHC II-restricted MAGE-A3 TCR on autologous CD4 T cells provided preliminary evidence of safety and efficacy [53].

##### (2) TSAs

TSAs are specific to neoplasms arising from oncogenic mutations, including genomic mutations. They are especially likely to occur as a result of key events in tumorigenesis (occurrence of single-nucleotide variations, indels, fusion genes, or chromosomal structural abnormalities), insertion and integration of foreign carcinogenic oncogenes (viral oncogenes) [94–97], or variants of events throughout transcription and expression of genes downstream (aberrant transcripts, aberrant post-translational modifications). TSAs are presented by MHC molecules and can be used to better characterize the heterogeneity of tumors and determine the therapeutic potential of TCR-T cells. In mutant cells, TSAs are also processed and transported by endosomal proteasomes to form p-MHC complexes with MHC-like molecules on the cell surface, whose epitopes are involved in receptor recognition. For immunodominant tumor TSAs, the sequential process of stimulating an immune response involves transcription, translation, and processing of the original peptide, presentation of the mutant peptide by the MHC molecule, the epitope of the pMHC complex, and the affinity of the TCR [98–100]. Thus, for the prediction and screening of TSAs, it is necessary to have full information about the types of MHC molecules possessed by the patient, and their genome and expression profile, as well as identification of the mutation and comprehensive follow-up analysis. Several shared neoantigens have achieved good results in clinical trials [21, 22, 101].

The major methods used to acquire TSAs are listed in Table 3. High-resolution mass spectrometry (MS) is commonly used to analyze samples and can be used in combination with a pan-HLA class I antibody for immunoprecipitation to capture tumor cell p-MHC complexes and determine polypeptide characteristics [102–105]. Jaeger et al. achieved precise extraction of a mouse-specific H2-K1-presenting peptide from in situ tumor tissues by inserting an inducible affinity tag within this MHC allele, resulting in a neoantigen that could not be predicted by mRNA expression or translational efficiency. This approach provided the TME and tissue-specific stimuli that are lacking from in vitro cell cultures, as well avoiding and confounding heterogeneous interference [106]. Whole-exome sequencing (WES) and RNA sequencing (RNA-seq) can be used as complementary methods to compare abnormal expression of tumor genes with that of normal genes and identify the sequences of mutated genes. RNA-seq can also be used to detect alternative splicing events and to estimate the relative frequency of mutant allele expression [107, 108].

**Table 3:** Acquisition strategies and main methods for neoantigens

Category	Introduction	Main method	Feasibility	Application scenarios
Direct acquisition of neoantigens	Sampling and screening of an existing population of tumor tissue, usually from biopsy or surgical resection Aim: to find available targets in their naturally occurring antigen pools	Immunoprecipitation-MS [102–105]	Patient tumor tissues	To capture primary tumor p-MHC
		Affinity-tag extraction [106]	Animal tumor tissues with specific MHC type tagged	To precisely extract known and neo-antigens in situ
		RNA-seq and WGS [107, 108]	Patient tumor tissues	To obtain complete serial sequence information of one patient
		peptide-MHC libraries [109]	Specific TCR or acquired T cells, and constructed vector libraries	To undifferentiately screen one TCR- recognizable known epitopes
		MANAFEST, T-SCAN [110, 111]	Specific TCR	To high-throughput screen recognizable epitopes
		SABR [112]	Specific TCR	To screen homologous epitopes
		Trogocytosis [114]	T cells fluorescently labeled with membrane proteins	To trace target cells binding and then sequence involved TCRs
		Hansolo system [115]	Patient T cells and immortalized B cell lines	To construct unbiased mutanome minigene recognizable library of the patient
Predictive modeling of neoantigens	Acquisition of patient's MHC molecular profile (individual-specific MHC typing) In silico analysis and prediction of deliverable epitopes combined with simulation of realistic multi-step parameter optimization, with attention to distortion or overestimation of the predicted epitope library Aim: capture of possible key antigens for usable TCR design	TCR and antigen prediction 1. Personalized information and MHC typing 2. Computerized prediction models: i. HLA typing ii. mutation typing and calling iii. HLA binding prediction iv. TCR prediction v. TCR priority vi. TCR-recognizing HLA screen [135] 3. Design of the corresponding TCR at the optimal epitope-MHC	Databases for in silico pre-analysis: • whole-genome sequencing and WES • RNA-seq • proteomics • MS	To predict epitopes and also exclude self-reactive antigens on a large-scale, use sequence information and select models

For TCRs for which concrete sequences have been determined by sequencing and manual design, undifferentiated screening of recognizable antigens can be performed using p-MHC homopolymer libraries constructed from baculovirus or yeast as vectors, with soluble TCRs as probes; this assists with the discovery of orphan TCRs, which have unknown antigen specificity in the natural state [109]. Mutation Associated NeoAntigen Functional Expansion of Specific T cells (MANAFEST) is a high-throughput platform that can screen known TCRs for recognizable neoantigens. T-SCAN is also a platform for homologous antigenic profiling of target T cells for large-scale genome-wide libraries [110, 111]. Signaling and antigen-presenting bifunctional receptors (SABR), a newly developed protein complex, recognizes a spectrum of homologous epitopes of specific TCRs [112]. Similarly, there are MHC-TCR double modified receptors that recognize antigenic targets of specific TCRs delivered by MHC-2 molecules on murine-derived CD4 T cells [113]. Notably, some new technologies have been used in antigen-searching biological toolboxes; for example, trogocytosis is a new T cell targeted ligand discovery principle that uses fluorescent labeling to trace membrane transfer of T cell membrane proteins to the p-MHC I of target cells, enabling isolation of the target cells and sequencing of the TCRs [114], as well as capture of orphan TCRs. Cattaneo et al. present HANSolo, a high-throughput system for unbiased p-MHC identification. In this method, the patient-matched Bcl-6/xL-immortalized B cell lines are modified for antigen-library expressing and specific T cell selection, which have all individual MHC genotypes [115].

Data from WES, RNA-seq, and proteomics in databases such as The Cancer Genome Atlas are used to initially screen for neoantigens across the cancer spectrum [116]. Databases have also been constructed with mature data accumulated from MS or from immunoprecipitation-MS or liquid chromatography-MS capture of antigens [117, 118], in conjunction with information obtained from NGS; these databases can be used for modeling and finding neoantigens, as well as for deep-learning-based prediction of peptide properties based on additional features such as liquid chromatography retention time, ion mobility, and MS/MS spectra [119].

Deep learning using databases and in silico prediction of possible sequences for obtaining and exhibiting neoantigens have become mainstream tools for neoantigen personalization. These require patient information related to tumorigenesis, infiltration, and metastasis, including genome, transcriptome, and proteome data, which can be compared with data from normal populations. The molecular properties of the MHC are polymorphic and diverse and influence binding to the TCR; the patient’s HLA allele determines the value and size of his or her tumor-specific predictive neoantigen pool [120–123]. Personalized information on specific patient treatments from NGS, WES, and RNA-seq, together with p-MHC acquisition by MS, can be used to estimate possible neoantigens that may arise, construct TCR–pMHC binding-prediction models, and estimate the ability of patients to affinity and transmit epitopes for specific MHC molecules. Machine learning enables the prediction of peptides from mutant homology libraries via a series of key steps. Filters must be added to the initial prediction outcome to exclude antigenic peptides that are not valid in the process. The filtering procedure involves parameterization of the delivery process of the antigenic peptide, including the affinity of the peptide for the MHC molecule, the sequence consistency of the variant peptide, the frequency and expression of the variant allele, and the capacity for peptide cleavage at the proteasome and subsequent translocation.

Some machine learning models identify mutations and predict neoantigens based on nucleic acid sequences; models with a focus on the identification of specific MHC molecules include NetMHC [124], NetMHCpan [125], and MHC flurry [126, 127], which consider the binding steps of specific MHC molecules but not the complex process of subsequent presentation. Some learning and prediction models use an HLA-ligand peptide dataset to improve prediction fidelity based on affinity; these include NetCTL [128] and NetCTLpan [129]. Experimentation is continuing with the introduction of more complex steps to train prediction tools and predict target TCRs that can bind to p-MHC and be recognized efficiently, for instance, McPAS-TCR [130] and VDJdb [131]. Although the feasibility of this approach has been demonstrated, the anticipated antigen pool may be exponentially larger than the actual antigen pool [132, 133], and most of the new antigens delivered by the predicted MHC molecules do not trigger an effective immune response [134].

#### Obtaining TCRs

**Fig. 2:**
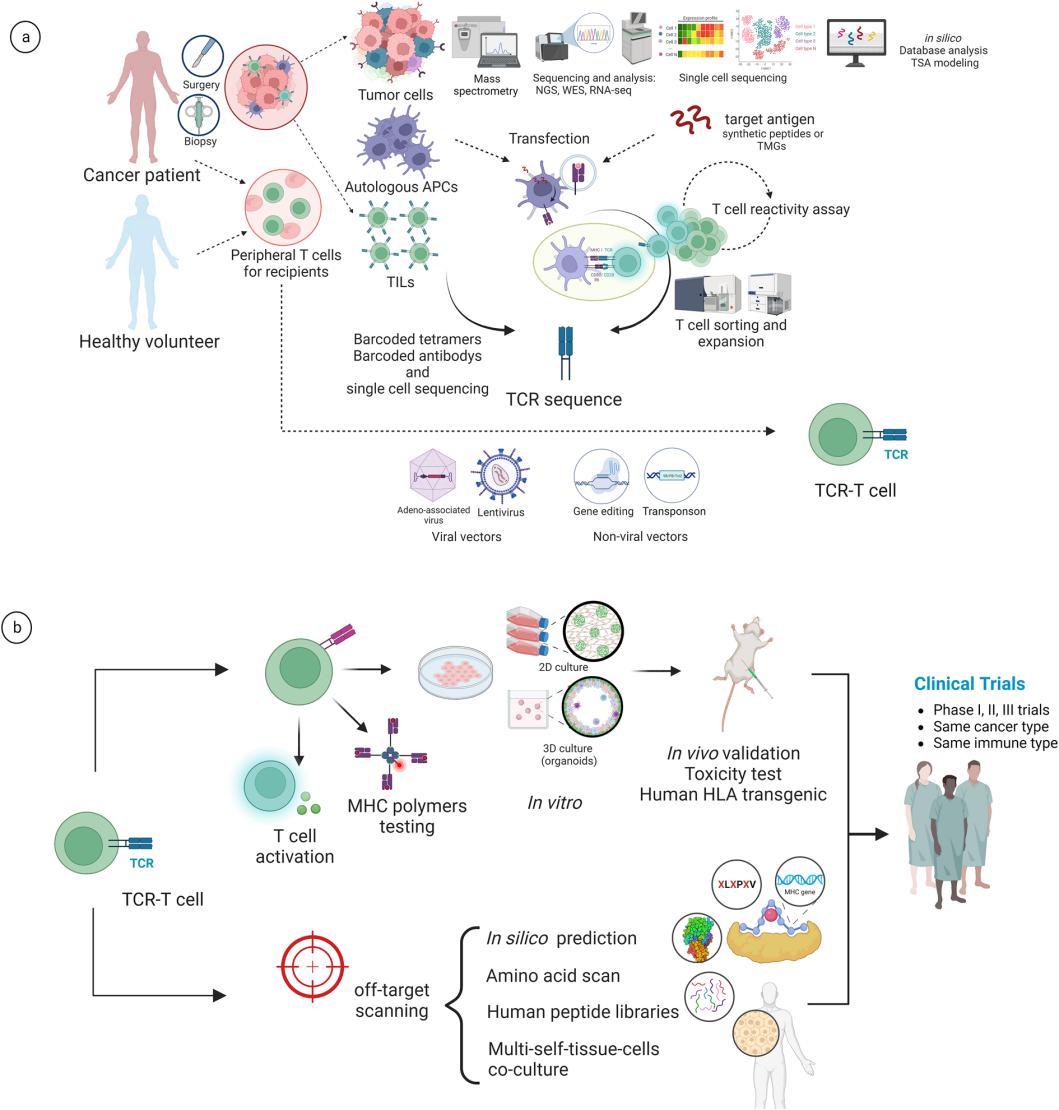
Overview of the necessary steps to develop, integrate, and test one TCR-T product. **A** Workflow for reforming recipient T cells to express TCRs that aim target antigens. First, target antigens are derived from tumor cells of individual patients. A series of protein acquisitions and multi-omics identifications are performed to identify tumor antigens, and new TSAs are computationally predicted. Acquired natural or synthetic antigenic peptides, or TMG transduction, enable autologous APCs to stimulate T cells with antigens to produce reactive TCRs or to detect T cell reactivity. Reactive productive T cells or in situ tumor-infiltrating cells are sorted and expanded, or screened using barcode tetramers or barcode antibodies, followed by single-cell RNA-seq or TCR sequencing, ultimately yielding the target TCR and gene sequence. Recipient T-cells can be obtained from peripheral T cells from the patient or from an HLA-matched healthy donor. A single tumor-responsive TCR-T is obtained by expressing TCR in recipient T cells via viral or non-viral vectors. **B** The effectiveness of a TCR-T can be validated by T-cell activity assays and MHC-polymer soluble ligands or a constructed libraries test. T cell activity is determined by antigen titration, HLA-matched cell line culture, and two- or three-dimensional (2D or 3D) tissue cultures. In vivo mouse models are established with human HLA for detection of tumor killing activity and toxicity documentation. Off-target activities of TCR-Ts on self-tissues can also be recorded simultaneously, via computer prediction, screening of reactive peptides for alanine or whole-amino-acid substitutions, screening of a full library of human self-peptides, and co-culturing of a variety of self-HLA-matched cell and tissue lines. Finally, TCR-Ts are subjected to clinical trials in various phases and several results have been reported

Sources of T cell clones include autologous T cells, derived from TILs within patients’ tumor or circulating T cells from their peripheral blood; and peripheral blood lymphocytes from healthy donors (Fig. 2A). The two differ in terms of T cell use and processing; autologous T cells are used for development of personalized TCR-T cell therapies, and some studies have also induced differentiation from hematopoietic stem and progenitor cells in vitro, progressing to production of mature single-TCR-specific T cells [136]. One p-MHC complex can bind to different TCR clones, and the specificity of the low affinity of TCRs for p-MHC allows one TCR clone to bind to a variety of different epitopes with low sequence similarity and different structures, as confirmed in recent studies. Owing to the simplicity and diversity of TCRs and the epitopes they recognize, identification of optimally specific TCRs in screening is complex; it may be necessary to take into account the immunogenicity of the corresponding antigens. The best TCRs with validated encoding genes can be sequenced by TCR sequencing, RNA-seq, and NGS to obtain coding sequences of both α and β strands and introduced into T cells via constructed vectors.

The strategy starts with T cell culture expansion using specific cytokines (interleukin-2, etc.) and T cell priming by co-culturing with antigen-presenting cells (APCs) loaded on antigen-homologous MHC molecules (incubation or gene introduction) to obtain specific T cell polyclonal populations. Assays of T cell proliferation, killing lysis activity by chromium release, enzyme-linked immunospot (ELISpot or FluoroSpot), intracellular cytokine staining (ICS) [137, 138], cytokine capture (IFN-γ), multiple intracellular staining, and activation or degranulation markers (41BB, CD107a) may be performed to detect the frequency, phenotype, and functional status of T cells [139, 140]. Alternatively, a fluorescent-protein reporter gene, coupled to the activation of the response element nuclear factors of T cells (NFAT), can be used to visualize characteristics of TCR-mediated activation. Fluorescence-activated cell sorting with fluorescent-labeled p-MHC tetramers, or MS sorting with heavy metal chelate-labeled p-MHC tetramers, can be used to screen antigen-specific T cells [141, 142]. Screening isolation of T cells can then be improved by various methods; tandem microgene (TMG) transduction or single-chain long peptide pulses on APCs is used to improve the screening efficiency of TILs at a higher order of antigen presentation [143, 144], although the expression levels of TMGs for the whole antigenic libraries may not be artificially controlled consistently [145]. DNA barcode-labeled p-MHC multimer- and tetramer-associated TCR sequencing has emerged as a method for high-throughput screening and precise TCR recognition of antigen-specific T cells [146–149]. Microfluidic technology has also enabled the dynamic detection and recognition of TCR–pMHC interactions at higher throughput [150, 151]; for instance, microfluidic antigen–TCR engagement sequencing technology allows high-throughput isolation and single-cell TCR sequencing of neoantigen-specific T cells [152]. In addition, a mouse model carrying a human-specific TCR has been constructed for the development and applications of high-affinity TCR [153]. The establishment of a heterologous immune system through mutagenesis of one of the TCR double-strands in the mouse thymus, or transfer of the entire human TCR αβ locus into mice to develop T cells targeting human self-antigens [154, 155], increases the probability of spontaneous occurrence of high affinity, without producing clonal deletion or tolerance of T cells as in humans [155].

Single-cell RNA-seq and single-cell TCR sequencing can be used to obtain phenotypic information of T cells carrying a single TCR clonotype, and targeted TCRs are obtained by sequencing TCR α and β single strands [156, 157]. High-throughput single-cell RNA-seq can be used to identify TCR transcripts from primed T cells [147], facilitating screening of early highly transcribed TCRs. Effective specific isolation of TIL in the unamplified state could avoid the problems of interference that occurs with high concentrations of IL-2 and deviation of the amplified state from the TIL polyclonal population [158]. Single-cell cellular indexing of transcriptomes and epitopes by sequencing (CITE-seq) and TCR-coupled TCR-CITE-seq can be used to target cell surface proteins of early TILs [159]. The new neoscreen platform was constructed to screen TILs early after specific antigen presentation and has achieved highly sensitive antigen-specific TCR isolation and identification [160]. Spindler et al. captured millions of natural TCRα/β clonotype libraries from primary T cells by massively parallel microfluidics processing and successfully constructed Jurkat cell lines for preservation, demonstrating that antigen-responsive TCRs can be screened with high throughput and specificity using a large-scale library [161]. The development of spatial transcriptomics has also enabled access to different phenotypes of tumor-infiltrating cells, as well as weighted localization, with applications in the definition of heterogeneity of metastatic tumors or tumors at multiple sites and in the search for shared immune cellular signatures. The newly developed Slide-TCR-seq method ensures fidelity and completeness of sequencing at scale of whole transcriptomes and TCR immunome libraries in the tissue environment, which has facilitated access to immunome libraries and enabled them to be compared with more sensitive and validated TCRs, including in different spatial contexts, even in a state of TME suppression [162, 163].

Progressively established decoy-RNA libraries targeting V and J regions of TRAC and TRBC pinpoint specific functional regions of specific segments of the TCR; they are used in pairwise deep sequencing of TCRs against oligoclonal populations of T cells [164, 165] and enable the identification of the full peptides of antigen-specific TCRs in human or human-derived mouse T cells. With single-cell sequencing information from T cells, Omer et al. applied a TRB prediction series pipeline based on IgDiscover, IgBlast, and TIgGER simulation software to multiple adaptive immune receptor repertoire sequencing (AIRR-seq) databases to retrace variants in the genetics upstream of the TCR haplotypes; this approach included tracing of unobserved coding genes, haplotypes, and loci, as well as mono-chromosomal or di-chromosomal deletions, and provided error-correction methods. This experiment confirmed the feasibility of using AIRR-seq information to resolve rearrangements and structural variants in the TCR V(D)J gene and to explain the germline variability caused by SNPs and changes in the expression profile of a specific TCR locus [166].

#### TCR editorial optimization

The original structures of TCRs can be modified artificially. Genetic engineering artificially creates affinity maturation of the complementation determining region (CDR); the highly variable CDR3 is important in peptide interactions and sequence diversity. Research efforts have been directed toward detection of this region and identification of its core motifs and basic features [167–169]. Several studies have been conducted on point mutation of CD3 loops for TCR affinity enhancement, which was subsequently shown to be effective and safe in clinical trials [42, 43].

For protein primary structure, the prediction of TCR recognition peptides can be optimized to induce targeted point mutations to alter their affinity for target antigens [170]. However, this involves artificial interference with the original negative selection result of the thymus, and attention to the emergence of non-target peptides for further identification is necessary. The sequence specificity of the predicted identified peptides can be determined using computerized deep learning methods. These include ERGO, which is based on a combination of McPAS-TCR and VDJdb, the two currently available large TCR–pMHC (I or II) datasets for training [130, 131, 171]. There are also post-training TCR and peptide binding prediction methods using natural language processing, in which candidate peptides are screened for specific identifiable TCRs [172].

Analysis of the shared motifs of TCR recognition core sequences has highlighted key conserved residues that drive TCR recognition, which can be used for further screening of TCR variants with core conservation [170]. This can also be achieved by peptide complex crystal analysis [173]. After identification of TCR conservation and specific target-antigen-MHCs, diverse mutant libraries, mainly on CD3α or CD3β, which have higher variability, can be constructed independently to obtain a larger population of TCR mutations. This involves mutation and enrichment of the remaining peptide recognition structural residues and flanks after retaining key residues [174]. A superior TCR with high affinity was obtained by screening for allogeneic p-MHC-guided T cell activity, and many mature TCR affinity engineering platforms have recently been constructed using mammalian cell lines [161, 175] or screening after precursor T cell differentiation [153].

Methods for protein structure training and model prediction are also being developed, with emerging strategies using modeling analysis or structure-guided analysis in three dimensions, and introduction of micro-interactions between interacting peptides for model optimization [176, 177], enabling more accurate prediction of recognizable protein responses. It has also been demonstrated that the reactivities of TCRs and MHC presenting peptides are equally affected by the structural diversity of p-MHC molecules [178]. Deep learning algorithms have been used for three-dimensional (3D) prediction analysis to train TCRs to recognize the binding effects of p-MHC [179]. Using AlphaFold, deep neural networks can predict TCR and p-MHC interactions, accurately distinguishing the correct available peptide epitopes, with applications in the development of generalizable predictive models for TCRs and further p-MHC-specific binding [180].

#### TCR-T cell construction

##### (1) Viral vectors

Once optimal TCR sequences and encoded genes have been obtained, they can be preserved or transferred by viral vectors into T cells suitable for tumor therapy. Adenoviral vectors were the first to be used for this purpose, although they are gradually being replaced owing to drawbacks such as their inability to integrate into the genome and encoding of heterologous proteins [181]. Replication-defective retroviruses, such as γ-retroviruses or lentiviruses, are now commonly used for the delivery of TCR target genes, and their stable delivery and safety have been demonstrated in human experiments [182]; however, the non-specific semi-random integration of their mechanism creates a risk of insertion of a random number of copies into the whole genome of the host cell [183]. This could interfere with the functioning of the transgene or even silence it [184], as well as posing unanticipated risks of other alterations in cytogenetic material. Adeno-associated viruses, which are widely used as vectors for gene therapy, can also be used for TCR-T construction, and good results have been achieved by combining them with CRISPR/CRISPR-associated protein 9 (Cas9) to achieve endogenous in situ knock-in (KI) of TCRs [185]. Recently, in an immunodeficient mouse model, Nyberg optimized an AAV synthetic subspecies, Ark313, for efficient transfection of murine-derived T cells; this could perform targeted transfer of large transgene expression cassettes with high efficiency, enabling nucleotide-free DNA delivery for CRISPR/Cas9-mediated gene knockdowns, with non-specific integration reported only as a rare event [186].

##### (2) Non-viral vectors

###### Retrotransposon system

mRNA electroporation allows for transient gene introduction and its use has also been reported for direct transient TCR and CAR expression. Clinical data to date indicate its efficacy and safety; however, this technique has limited durability for a single safe transfer of a certain amount of TCR, and its efficacy is restricted. It is currently used to introduce other transgenic systems. These use transposons that are flanked by terminal inverted repeat (TIR) sequences and contain DNA double strands of transposase-coding sequences; there are also semi-autonomous transposon systems that can be supplemented with the expression of transposases in the form of synthetic plasmids and mRNAs, enabling artificial control of transposon DNA expression [187] Such transposases are highly specific [188], ensuring to some extent stability and safety after transfer.

The Sleeping Beauty (SB) transposon was originally derived from an inactive copy of a DNA transposon of the Tc1/mariner superfamily [189] and has TIRs of approximately 230 bp at both ends, with internal sub-structural domains including a nuclear localization sequence, DNA-binding structural domain, and catalytic structural domain [190]. SB is now widely used in ACT, and its powerful transduction ability enables the transduction of long sequences of up to 6000 bp into mammalian cells for double-stranded cleavage and insertion. The integration profile of SB in mammalian genomes has been shown to be near-random in clinical trials; this reduces the risk of insertion mutagenesis [191–193]. SB has been shown to be an effective and safe approach for introduction of specific receptor genes into human T cells [194–196]. In CAR-T cell therapies, in particular, its applications are relatively mature, and it can be used to introduce transgenic CAR particles. A recent study combined CRISPR/Cas9 technology with knockdown disruption of alloreactive TCRs and subsequently reduced homozygous reactivity, thereby providing an alternative means of inactivating donor TCRs as a universal source of T cells. CD19 CARs were imported using SB and maintained stable and potent expression [197]. There have also been initial attempts to conduct a quantitative production process for CAR-T based on SB, illustrating that the efficiency of transposon systems for widespread production can be increased [198]. These valuable applications also contain lessons for further applications of SB in TCR-T cells.

The PiggyBac (PB) transposon system uses a transposon originally from insects, which is 2475 bp in length and contains one transposase-encoding open reading frame, encoding dimerization, DNA-binding domains, a catalytic domain, an insertion domain, one N-terminal domain, and one cysteine-rich C-terminal domain [199–204]. PB has been demonstrated to be active in in vitro assays and in yeast, mouse, and humans [202, 205]. The integrated PB tends to be inserted into a TTAA sequence, which is subsequently replicated and inserted into the transposon flanking sequence [206]. High efficiency has been achieved for gene transfer of PB as a vector in CD19-CAR-T cells [207]. PB is also available as an alternative to the non-viral vector approach for TCR-T gene introduction [208]. A recent study demonstrated the presence in the human genome of a homologous protein, PGBD5, derived from domesticated PB transposons [209], suggesting possible cross-reactivity between the PB endogenous human transposon and a risk of cross-reactivity of exogenous sequences. The applications of PB and the related clinical progress in CAR-T cell therapies for solid malignancies in recent years are worthy of note [210].

Tol2, the only transposon identified from vertebrates with autonomous transposition activity, contains incomplete TIRs of 17 bp and 19 bp and three subterminal repeats of ~ 30 bp near the right TIR and is capable of sustained transgene expression after gene delivery. In contrast to that of other transposon systems, the transgene efficiency of Tol2 is well stabilized in mouse strains and human systems and is not affected by endogenous factors such as gene silencing mechanisms in mammalian hosts [211–215].

The transposon types of listed above possess relatively low immunogenicity and a small genomic footprint; however, the gene transfer efficiency of various transposons varies from target cells, and one of their distinctive features is that their efficiency is negatively correlated with the size of the transposon expression cassette. In the context of proven efficacy and safety in ACT, large-scale transposon transfection with guaranteed efficiency is key to dissemination; however, the negative aspects of the randomized nature of the integrated genome should not be overlooked [195, 216].

###### Gene editing technologies represented by CRISPR/Cas9

Gene editing with CRISPR and the Cas9 endonuclease is emerging as a precise means of introducing exogenous TCR genes, enabling flexible reprogramming of TCR-T cell signaling.

Early milestones in gene editing were achieved using short interfering RNAs, ZEN, and TALENs to silence PD-1 or endogenous TCRs, or to create antigen-specific artificial T cells [217–220]. The superiority of CRISPR in precisely targeting multiple genes at different locations using guide RNA sequences allows for simpler preparation and target library expansion [221]; however, there is a relatively high risk of genetic modification toxicity and off-targeting according to clinical reports of ACT therapies [222]. Such gene-editing systems can be introduced into target cells using lentiviral or AAV vectors. Then, single-guide RNAs are used to fine-tune the DNA sequence target, and Cas9 proteins cleave the DNA to produce double-strand breaks (DSBs), followed by classical autonomous repair of host DNA by non-homologous end joining (NHEJ) and homology-directed repair (HDR) guided by the HDR template (HDRT) [223]. NHEJ, which usually involves a direct joining of DNA break ends, can be used for T cell knockout (KO); however, this simple repair process can lead to the introduction of new insertion mutations and interference with gene function [224]. Instead, targeted T cell KI by HDR can be achieved through design and addition of exogenous HDRTs. The current preference is for the transfer of the HDRT into cells to be accomplished using single-stranded DNA donors and virus-independent gene electroporation, as this method has the advantages of ease of production, low cytotoxicity, and safety [225, 226]. However, the effective acquisition rate of HDR CRISPR individual gene KIs reported by Roth et al. was not high, and the harvesting of KI homozygotes in KI double strands was challenging [226]. Several protocols have been developed to improve HDR KI efficiency, including tuning of the parameters of the T cell introduction program and optimization of the HDRT design to improve nuclear translocation efficiency and KI efficiency [225]. Another study proposed a new way to improve the efficiency of HDR KI by intervening in the choice of repair mode after targeted editing and inhibiting the bypass of NHEJ repair by adding small-molecule interfering agents [227]. However, the T cell status and corresponding editing procedures need to be closely monitored and adjusted during the process of T cell editing using CRISPR/Cas9, so that the relative efficiency can be improved and optimal transformation and survival can be achieved [228, 229]. Finally, the composition, functional integrity, and genetic information of the product should be confirmed and verified.

The main advantage of using CRISPR/Cas9 in ACT is that it enables precise editing of multiple loci simultaneously [230]. This allows for the precise introduction of engineered receptor genes and simultaneous KO of endogenous-related genes, such as endogenous TCR genes and T cell suppressor genes [23], resulting in streamlined production. Knocking tumor-specific receptors into the endogenous TCR constant motifs TRAC and TRBC by CRISPR/Cas9 has been shown to effectively improve the killing activity of gene-edited T cells [231]. The effectiveness of the obtained CAR-T [232] and TCR-T cells for the treatment of hematologic malignancies, some melanomas, and solid tumors such as synovial sarcoma has also been preliminarily demonstrated in mouse models [233, 234] and human clinical trials [235–237].

Stadtmauer et al. reported the first human phase I clinical trial of CRISPR-engineered TCR-T cells. Using CRISPR/Cas9 for multiple gene editing of T cells intended for therapeutic applications in three patients with refractory solid tumors, they introduced a specific artificial TCR NY-ESO-1, which simultaneously knocked out both the endogenous TRAC and TRBC genes to reduce mismatches, as well as supplementally knocking out the third gene, PD-1, to comprehensively enhance the anti-tumor activity of the T cells. Their study proved the in vivo feasibility of CRISPR gene editing for TCR modification in the clinic and the lasting nature of the modifications, and it has already been approved by the regulatory authorities as the first human safety study [238]. Subsequent phase I clinical trials have likewise confirmed the efficacy and promising long-term functionality of NY-ESO-1-specific TCR-T cells for refractory synovial sarcoma [45]. Recently, Foy et al., using a non-viral CRISPR/Cas9 editing approach, knocked out both the TRAC and TRBC genes in one single step and inserted two strands of a neo-TCR derived from a patient PBL into TRAC loci. In a phase I trial (NCT03970382) in 16 patients with refractory tumors, treatment was successful: five patients remained stable in terms of disease progression, whereas the remaining 11 showed a good response to the therapy [23]. Parallel knockdown of in situ TCRs is now routine practice, and the benefits of this are discussed in detail later. Notably, Stenger et al. in a summary study of various ACT editing methods, found that retention of endogenous TCRs resulted in significant improvement in T cell persistence compared with endogenous TCR retention when using TCR-KO-anti-CD19 CAR-T cells for the treatment of human patients with CD19 + leukemia [239, 240].

To reduce the risk of uncontrolled proliferation and toxicity of ACT cells, a number of strategies have been developed to set a start switch for small-molecule drugs such as rituximab or sirolimus [241, 242]. CRISPR/Cas9-mediated introduction of suicide genes has likewise been used to achieve switch-off of the functions of engineered T cells [243], ensuring their controllability and subsequent use in clinical applications.

More recently, studies have aimed to increase engineered T cell yields and enable scaled-up T cell editing by combining the newer homology-independent targeted insertion approach to DNA repair with the CRISPR/Cas9 system [244]. High-fidelity Cas9 proteins showing superior efficacy, precision and a shorter period of editing activity, in many Cas9 enzyme systems could create more favorable conditions for optimizing Cas9 in the clinical design of future engineered T cell products [245, 246]. In addition to NHEJ- and HDR-mediated CRISPR/Cas9 modifications, DSB and HDRT-free base editing are rapidly evolving. Base editing has been shown in preclinical studies to reduce the off-target and chromosomal translocation risks associated with earlier methods without inducing DSBs [247]. Webber et al. also achieved multiple base editing of the T cell genome using mRNA electroporation, with high efficiency in simultaneous editing of multiple T cell loci [248]. However, there is a risk of complex genomic alterations or rearrangements potentially resulting from lentiviral transduction or DNA-based delivery of CRISPR/Cas9 systems [249]. By contrast, Cas9 ribonucleoprotein delivery systems for T cells have advantages including greater editing efficiency and less toxicity [250, 251].

## Applications and constraints of TCR-T cells

### Identification and applications of TCR-T cells

#### Evaluation of TCR affinities and recognizable epitopes

Molecular bioinformatics pre-assessment is used to elucidate the affinity process of target TCRs and clarify kinetic and cytological parameters, followed by a combination of computational scanning and experimental techniques to perform ultra-high-scale screening and save time in preclinical studies. The use of a computer-based in silico approach allows for the pre-modeling of large-scale libraries of TCR-recognizable epitopes, although specific p-MHC binding remains difficult to predict. The computational approach can also incorporate sequencing information for the construction of core sequence libraries [252].

Affinity prediction can be based on a deep-learning approach that predicts the immunogenicity of peptides and determines key residues for T cell recognition, as well as simulating the physicochemical properties and immunogenicity that define the corresponding real-world conditions [253]. Enhancing the recognition of tumor antigens by increasing TCR affinity has been of clinical interest [254]. High-affinity TCR-modified T cells can detect lower levels of tumor antigens, do not rely on the adjuvant role of CD8 co-receptors, and can produce MHC-1-restricted CD4 T cells to secrete positive cytokines and promote immune initiation [255–257], contributing to the tumor-suppressive milieu and the diversification of TSAs. However, there is a concomitant risk of high-affinity TCRs recognizing and attacking normal tissues.

Targeted TCR affinity sorting and affinity maturation based on the binding of a given antigen are usually performed using yeast display and phage display technologies; these, combined with the soluble tetrameric, dimeric, and monomeric p-MHC ligands, can be used in high throughput to screen natural high-affinity TCRs, identify libraries of TCR mutants with modified affinity, or validate the affinity of TCRs for specific antigens [167, 258–261]. A library of stably expressed TCR sentinel mutations can be obtained, and soluble p-MHC ligands can be prepared using high-throughput sorting techniques. However, these methods are unable to regulate and predict the complex specific binding or cross-reactivity of antigenic peptides [262]. In addition, the protein expression and modification capabilities of the cells used to display the libraries remain limited [258], potentially causing distortion of antigenic peptides.

The effective activation of p-MHC cannot be characterized based on TCR binding alone. It has been consistently found that high-affinity TCRs strongly bound to p-MHC do not elicit agonist-stimulated interactions [263]. Using a p-MHC yeast library and soluble TCRs to identify collected inactivating TCRs, the “catch bonds” at the TCR–pMHC binding interface have been defined using molecular dynamics simulations of the TCR–pMHC binding interface, and the formation and persistence time (lifetime) of this force have been shown to be was positively correlated with the functional potency of TCR–pMHC-associated interactions [264–266]. Follow-up studies demonstrated that this positive effect results from mechanical-force-induced conformational changes in p-MHC that enhance pre-existing contacts and activate new interactions at the TCR–pMHC binding interface to resist force-induced bond dissociation, leading to formation of TCR–pMHC catch bonds and activation of T cells; moreover, the balance of such conformational changes is correlated with the isoforms of HLA molecules [267]. Such force bonds have been used to assist in the identification of TCRs with strong activity and high specificity for specific HIV-Pol and MAGE-A3 antigens, enabling refinement of the design and functional screening of TCR libraries for structural characterization [268]. The opposite “slip bonds” are thought to reverse the force action of TCR and p-MHC interaction and down-regulate the bond lifetimes; their rupture under external forces leads to inactivation of homologous TCR recognition of specific peptides [264]. Further, in biophysics, the cell surface TCR is understood as a multi-module mechanosensor that is force-sensitive to the recognition module of a moving p-MHC, where dynamic recognition of the bond is instantly transmitted to the non-directly covalently associated TCR signaling module. Physical alterations, such as molecular deformation, enhance or attenuate this non-covalent binding and prolong the bond lifespan, and the recognition of an antigenic peptide by a TCR is understood to be the result of the bond’s immunogenicity. Immunogenicity of TCR-recognized antigenic peptides is understood as an alteration of the bond [269]. However, these two bond-based explanations for the diverse affinity differences in the ability of TCRs to turn on, hold, and turn off the force of p-MHC recognition remain complex and open to refinement. Recent findings suggest that in non-cellular molecular experiments, low-affinity TCR–p-MHC pairs with faster solution off-rates have external force insensitivities that are more resistant to mechanical forces (weak sliding or capture bonds); i.e., low-affinity TCRs improve their retention of recognition for antigens [270]. In addition, covalent TCR-pMHC interactions such as disulfide bond formation can occur secondarily, enhancing the interaction and activating TCR ligand-signaling T cells with limited affinity [271]. Therefore, current methods for screening TCRs may lead to unanticipated deviations between predicted functions and the natural state, and the real impact of their affinity on T cell actions needs to be further verified and corrected.

TCR affinity and activity are not always correlated; TCRs with high antigenic affinity (1–5 μM) tend to exhibit high activity in vitro, and TCRs with low to medium affinity (5–100 μM) usually show poor correlation between affinity and activity [264]. Therefore, the actual responsiveness of TCRs to the target p-MHC still needs to be assessed. A recent paper proposed using DNA origami technology for exploration of the complex problem of T cell sensitivity to p-MHC, for instance, in the case of TCRs with medium/low affinity, those present in small amounts, or even individual p-MHC agonists. This platform would enable precise intermolecular nanoscale microscopic distances to be determined on simulated APC membranes to achieve quantitative control and localization of TCR populations on the surface of individual T cells, as well as bio-interfacial mimicry for recognition and binding of p-MHCs [272].

#### Adverse event reporting

In clinical trials, artificial TCR-T cells have shown unanticipated post-administration cross-reactivity in humans, with fatal effects in some cases. Objective responses were observed in one-third of cases in a clinical trial of TCR-engineered T cells targeting CEA, with all three patients who received the drug experiencing severe post-treatment transient colitis as a side-effect [90]. Two early concurrent independent clinical trials of engineered high-affinity TCR-T cells targeting MAGE-A3 showed considerable neurological and cardiac off-target toxicity. One of these, using T cells with CD3 regionally directed mutagenesis of mouse-derived TCRs, showed neurotoxicity along with complete remission of clinical outcome in five of nine patients, and two cases of post-treatment brain death, the cause of which was later shown to be recognition of normally expressed MAGE A12 in the brain by the MAGE A3 high-affinity TCR [52]. Another experiment was terminated prematurely after participants suffered cardiogenic shock and there were two post-treatment deaths [51]. Subsequently, the affinity-modified TCR-T with an artificially engineered mutation in the CDR2 region produced cross-reactivity against titin in normal cardiomyocytes, resulting in cardiotoxicity [273]. Notably, these proteins exhibit a high degree of homology with the MAGE family. These two reports provide warnings, as well as ideas for subsequent target prediction development and cross-reactivity assays. Detection of TCR and peptide binding alone is not sufficient; detection of T cell activation can provide a more accurate (and more intuitive) assessment of TCR specificity and cross-reactivity. Negative selection of TCRs for specific peptides does not exclude their effects with homologous mutant peptides.

#### Rehearsing and ruling out off-target effects

When TAA-targeting TCRs are used to target tumors, TCR affinity above a certain threshold will recognize target cells and initiate cross-talk signaling owing to low levels of expression of these antigens or cognate antigens in normal tissues. Therefore, the actual TCR affinity must be controlled to remain below that threshold and tested for its strength, to avoid undermining the required tumor selectivity. When TCRs are evaluated, the sequence identifying the target antigen or specific epitope is known, and epitopes are initially mutated and evaluated using alanine scanning or full amino acid scanning for amino acid point mutations at each position outside the anchored position, which usually remains a 9-mer or 10-mer conserved core motif [273, 274].

Furthermore, human endogenous peptide databases can be scanned using epitope-wide all-amino-acid point mutations to screen for existing self-TCR-recognizing epitopes and potential cross-reactivities [275]. However, the relatively conservative alternative constructs result in antigenic display libraries of limited practical size, which may not be sufficient for the required exclusion. Further, there are artificial methods of screening for possible TCR reactions using yeast libraries [109, 276] or combinatorial peptide libraries [277], and extensive computerized search rules through combinatorial peptide libraries have been established and evaluated [278]. TCR interactions can be tested against self-tissue libraries to exclude possible off-target side-reactive antigens, and TCR fingerprints are confirmed by TCR reactivity to antigenic libraries corresponding to TCR sequence specificity [279]. TCR fingerprinting can be used to identify potential target epitopes for the presentation of specific MHC-1-like molecules, or in reverse to screen for the best-reacting TCRs for specific antigens. Research has confirmed the uniqueness of TCR fingerprints, which use the characteristic pMHC motifs of each individual TCR as intrinsic features of TCRs [279, 280]; these fingerprints can be used for fine differentiation and prioritization in the search for TCR clusters that recognize the same pMHC clusters. The universal T-SCAN platform for positive screening for tumor epitopes can likewise be used for cellular presentation and exclusion of self-reactive antigens at higher throughputs, reducing the risk of cross-reactivity in the context of large self-antigen libraries [110]. Recently, bioinformatics analysis of protein sequence spatial landscapes has allowed for position-specific amino acid preferences to be complemented by conformation in the TCR–pMHC-bound state, accelerating the detection of potential homologous protein cross-reactivity trends [252]. Experimental identification and estimation in silico of off-target reactivity of TCRs against specific tumor antigens is usually performed on a large scale using MS-based immunopeptidomics [83, 281].

#### Multiple characterizations of TCR-T cells

Immunological studies titrate homologous peptide antigens to estimate the affinity of TCR-T cells and assess their relationship with peptide concentration. Cytological studies are conducted to measure the response of “target immune cells” to a set of HLA-typing-matched progenitor cells in co-culture and to detect the activity of T cells (such as proliferation, cytotoxicity, and release of cytokines) in response, as described in the previous section. A new method uses peptide library APCs to express anti-cytokine antibodies and identify defined HLA molecules and peptide epitopes recognized by orphan TCRs by detecting the secretion of specific cytokines IL-2 and IFN-γ from T cells, as well as converting binding interactions into universal signals for different types of HLA molecules (not limited to CD4 and CD8 T cells) to identify peptide libraries to be detected on a scale of thousands of peptide oligonucleotide libraries; this scaling up has a lower cost compared with ELISPot and ICS [149]. Cells and cell lines to be tested should include tumor cells, autologous cells, and HLA-diverse lymphocytes for in vitro validation of tumor killing activity, autologous reactivity, and allogeneic reactivity of engineered TCR-T cells [282] (Fig. 2B). Histologically, cells are cultured in standard cultures or organoid cultures using two-dimensional (2D) or three-dimensional (3D) materials, and tissue cell phenotypes are characterized by immunohistochemistry to detect T cell reactivity. The use of 2D and 3D materials in vitro to mimic organoid environments can contribute to a better understanding of the cell-to-cell actions of TCR-T cells. Joseph et al. cultured cardiomyocytes, astrocytes, and endothelial cells, as well as terminally differentiated human cells derived from induced pluripotent stem cells, as normal cells to observe T cell reactivity, and an immortalized B-cell line, B-LCLs, to characterize the alloantibody response. They also used 2D and 3D materials to observe the killing effect of TCR-T cells on microtissues [274]. To ensure autoantigen coverage, human cells can be subjected to specific events to expose certain potential epitopes, for instance, treatment with IFN-γ to induce immunoproteasomes and replication of immune peptides exposed during inflammatory events [274].

Detection of antitumor activity in vivo begins with a mouse xenograft model of tumor cells, which should be constructed and used according to the Scientific Procedural Approach to Animal Testing, with the regulatory authorities FDA and EMA supporting the 3R principle [283]. Construction of an immunodeficient xenograft mouse model can be used to confirm TCR-engineered human T cell efficacy; however, toxicity assessment in this model is limited by the lack of HLA molecules in the host and the poor persistence of human T cells in mice [284]. Reliable in vivo models to assess TCR cross-reactivity and allogeneic reactivity are still being developed. The T2EVOLVE Consortium, a public–private partnership, describes currently available preclinical models, tools, and specifications for TCR-T cells and their clinical safety and efficacy [285]. Innovative animal models for TCR-T cells have been created that are more in line with desired characteristics, such as the humanized SGM3 model used to more sensitively discriminate subtle/nuanced differences between ACT cells produced from different starting cell sources [9]. Studies of antigen-specific TCR-T in patients with solid tumors have been carried out in multiple phases of clinical trials, and lymphocyte clearance of drugs such as cyclophosphamide and fludarabine is usually applied earlier in clinical trials to achieve better T cell implantation, proliferation, and persistence [286, 287]. However, lymphocyte depletion predictably increases the incidence of several hematologic toxicities (neutropenia, anemia, and thrombocytopenia) and infectious complications, with consequent increases in the incidence and severity of cytokine release syndrome (CRS) and, in some cases, tumor lysis syndrome [288]. The solid tumor response of patients can be documented according to the Response Evaluation Criteria in Solid Tumors (RECIST ver. 1.1) [289]. Some modified and proposed versions such as immune-related RECIST (irRECIST) [290] and immune RECIST (iRECIST) [291], have been not standardized in evaluation of TCR-T clinical trials.

### Existing constraints and approvals

#### TCR α/β chain mispairing

**Fig. 3:**
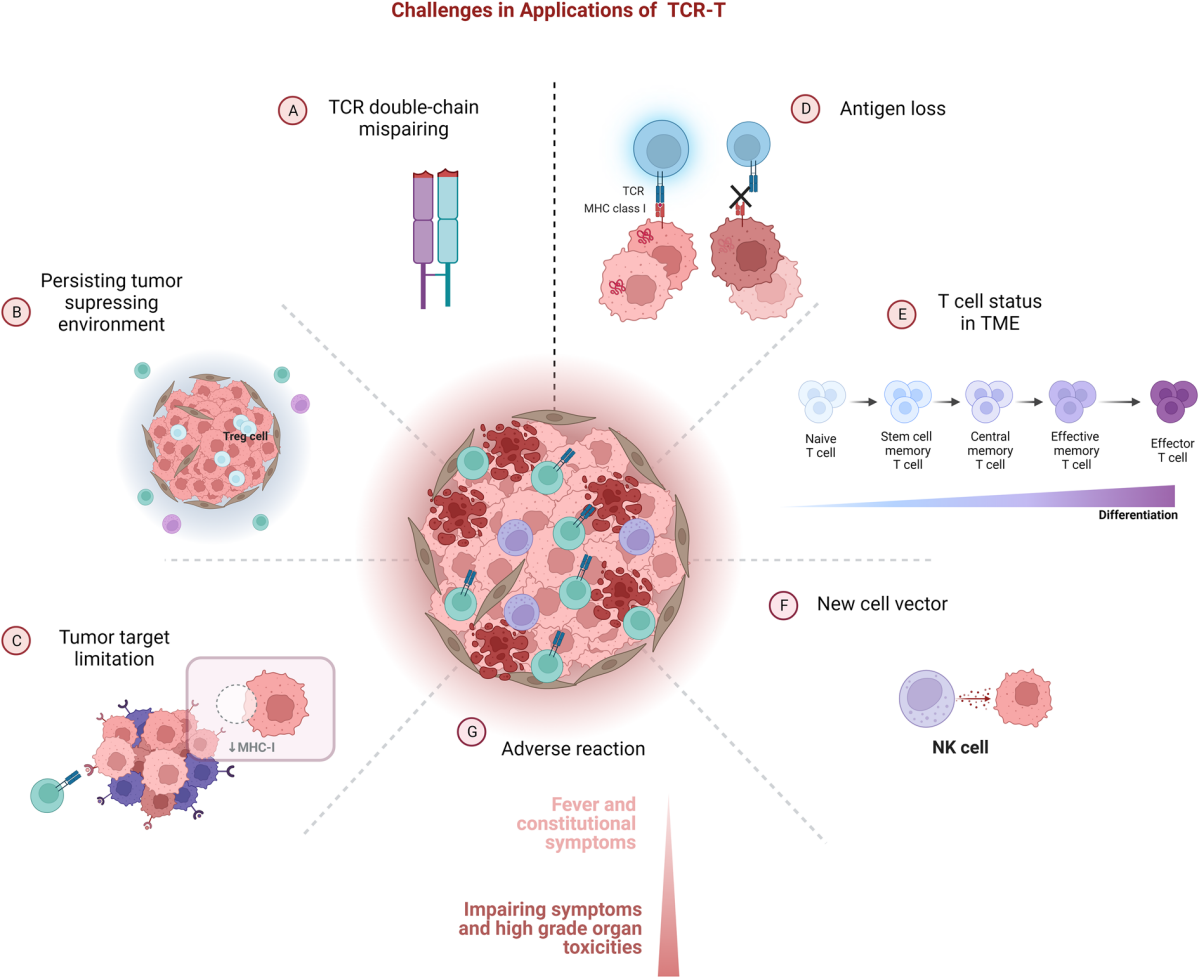
Challenges in applications of TCR-T immunotherapy for tumors. Applications of TCR-Ts in tumor immunotherapy still have some limitations and potential improvements: **A** Mispairing of introduced TCRs and endogenous TCRs may occur on TCR-Ts. **B** Multiple factors contribute to an immunosuppressive TME. **C** Antigenic heterogeneity of solid tumor tissues and decreased MHC-I molecules pose obstacles to TCR-T recognition. **D** Antigenic drift and loss in tumor cells occur during solid tumor development. **E** T cells are heterogeneous in different states of differentiation, with respect to characteristics such as activation capacity and lifespan. **F** Applications of natural killer (NK) cells or natural killer T cells as vector bring new advantages and possibilities. **G** TCR-Ts have demonstrated numerous adverse effects in clinical trials, most commonly cytokine release syndrome

Endogenous TCR-encoded TCR α/β chains continue to be expressed intact [292]. As endogenous and transgenic TCRs are simultaneously expressed, they may heterodimerize to generate four distinct TCRs and persist in the circulatory system. Simply introducing exogenous TCR genes upstream of the host expression set and additionally increasing TRAC and TRBC expression will result in the coexistence of endogenous TCRs, exogenous TCRs, and heterodimerization with endogenous and transgenic TCRs [292] (Fig. 3). TCR heterodimers, owing to their unselected and unscreened thymus, may be of unknown immunogenicity and auto-antigenic reactivity, and the infusion may trigger an auto-crossing immune reaction. This reaction may even be fatal, for instance, graft-versus-host disease (GVHD) demonstrated in mouse models [293, 294], although GVHD-like toxicity has not been documented in patients receiving TCR-T products to date [295, 296]. In addition, coexistence of multiple TCRs can lead to competition for limited CD3 downstream molecules and co-stimulatory signals, reducing the efficiency of the target TCR.

Treatment for such mismatches has been systematically established to reduce the structural similarity between exogenous and endogenous TCR single chains and increase the structural specificity of the engineered α/β chains to match. The first method for modification of exogenous TCRs involved modification of the extracellular constant region. For example, the residues in the constant region of human TCR were partially or completely replaced with the constant region of mouse TCR. A human immune response against the TCR region from mice does not impair the efficacy of T cell therapy or increase the body’s additional response. Mouse TCR constant region replacement may enhance the safety and functionality of TCRs [86]; however, further observation of its use in TCR-T cell therapy is required, as the murine modification has been found to affect the anti-CD19 CAR effect in clinical tests [297]. Modification of TCR residues has also been achieved by the introduction of two complementary cysteine residues, adding additional pairing of the engineered TCR α and β chain disulfide bonds [298], by replacing the TCR α chain TM region with hydrophobic residues to stabilize its expression [299], by co-expressing a TCR single chain fused to CD3ε [300], or by using structural domain inversion or swapping of the constant domain of TCR double strands to minimize mismatches [301–303].

KO or silencing of endogenous TCRs can also be considered as a means of reducing the incidence of mismatches. This can be assisted by additional modules such as RNA interference [304] or KO of endogenous TCR loci with CRISPR/Cas9, which is a more precise method and an easier one to use. KI of the target TCRs at the location of the original locus saves processing and effectively achieves the engineered TCR dimer pairings; this approach has already been tested in clinical trials, as described in the previous section.

#### Adverse reactions

Owing to low levels of TAA expression in normal tissues, introduction of exogenous TCR-T cells may cause cross-reactivity elsewhere in the body. For example, severe events were reported in the two MAGE A3 TCR-T cell clinical trials, where the homologous antigen MAGE A1 was expressed in ocular or cutaneous melanocytes. In addition, in clinical trials of TCR-T cell therapies targeting MART 1, patients treated with the therapy developed unanticipated ocular uveitis and hearing loss [35–37]. Significant adverse events on MAGE-A homologous antigens emphasize the exclusion of potentially reactive antigens of candidate TCRs within the whole space-wide proteome and the elimination of autoimmune cross-reactivity. TCRs should recognize peptide with an identical epitope sequence, furthermore, may recognize structurally similar peptide-HLA class I complexes despite differences in peptide sequences. Some means of removing excess T-cell toxicity, such as inducible suicide gene introduction [243], are also worthy of consideration.

CRS is the most common and serious immune-related adverse event encountered in recent clinical trials of engineered T cells and mostly occurs within 14 days of ACT infusion [36]. Symptoms of CRS range from mild fever to life-threatening symptoms and multiple organ system failure, and include headache, encephalopathy, tremors, and seizures. CRS is currently thought to be associated with T cells or to involve immune cell activation and secretion and tumor cell lysis, which is related to the variability in levels of antigen expression among patients. Moreover, immune effector cell-associated neurotoxicity syndrome, often referred to as neurotoxicity, is very common, especially in patients receiving CD19 CAR-T cells [305].

Although CRS in ACT is strongly associated with elevated levels of ILs such as IL-2, IL-6, IL-5, IL-8, IL-10, and of TNF-α, and some of these cytokines have synergistic effects on T cell activity and lifespan, the IL-6 receptor antibody tocilizumab is currently considered to be a suitable option for drug control [306]. Attempts to improve transgenic T cells are also underway, and it has been found that spatial site-blocking effects can be attenuated over time; in situ polyethylene glycol affixed to the surface of CAR-T cells slows monocyte activation and effectively attenuates CRS symptoms and neurotoxicity [307].

#### Limited therapeutic effect

One of the main features of refractoriness in solid tumors is the easily acquired T cell resistance of the tumor tissue and mesenchyme, the causes of which may be primary or secondary. However, the impairment of T cells is regarded as the result of a complex network of negative signaling interactions.

Primary resistance arises from tumor antigenic characteristics, including antigenic expression drift, heterogeneity, or low antigenic expression [308], especially in cancers carrying high mutational loads. The genetic heterogeneity in the targeting of TCRs to some limited neoantigen also leads to functional limitations of TCR-T cells that accompany this heterogeneity, analogous to the inactivation of ICIs [309], and the tumor epigenetic heterogeneity of patients also generates antigenic variants and generates new TCR tolerance [310–312]. Thus, the need for shared antigenicity has been emphasized during TCR development, in order to provide deep coverage of tumor tissues in both spatial and temporal dimensions, better selection of early key antigens with less susceptibility to antigenic drift is needed, as well as an emphasis on achieving broadness in the individualization of the antigenic screening process, in a single dose or in phases, by administering therapies targeting multiple target antigens.

Secondary resistance is triggered extrinsically by the tumor after T cells have been equipped with a specific TCR library. Arising from the evolved immune escape of tumor cells, it resembles the natural pathway of immunosuppression of T cells by the TME and features a variety of molecular mechanisms. Its most direct manifestation is the downregulation or loss of MHC-1-like molecules and altered expression of corresponding immune factors. Downregulation of MHC-1-like molecules prevents the effective presentation of target antigens, or the suppression of co-activating signals and activation of co-suppressive signals. Impairment of natural T cell function also occurs via restriction of dendritic cell (DC) migration towards the draining lymph nodes, or through inhibition of the antigen cross-presentation process in paracrine cells [313, 314], which may also interfere with the sustained activation state of the engineered T cells. A recent study identified a new axis of T cell killing action in tumor cells escaping from MHC1 downregulation [315]—natural killer group 2D ligand (NKG2DL) and T cell NKG2D interaction. During classical tumor escape by downregulation of MHC molecules and antigens, CD8 T cells maintain their killing capacity [315], suggesting that it is beneficial to maintain or enhance the original natural killing pathways and new targets of T cells for the maintenance of TCR-T cell activity. Moreover, to enhance intracellular TCR signaling to improve T cell activity under existing conditions, simple general modifications in the variable region of the TCR have been found to increase levels of cell surface expression of the TCR. Three amino acid residue substitutions in the framework of the variable structural domain of the TCR effectively enhanced TCR recognition ability and TCR-mediated proliferation and secretion of killer cytokines from T cells [316].

#### T cell exhaustion

Clinical and infusion of TCR-T cells has demonstrated that these engineered T cells can generate a memory phenotype and maintain long-term survival in vivo [317, 318]. However, T cell depletion, defined by a progressive decline in T cell function due to the presence of the TME, continued exposure to tumor antigens, and TCR stimulation, may lead to transition to a state of terminal depletion [319], characterized by TCR signaling co-suppressor receptors such as classical PD-1, CTLA4, and LAG3 [320]; active immunosuppressive enzymes such as CD39 [321, 322]; or the expression of intracellular negative factors that downregulate the intracellular cascade of responses in which the TCR is involved [323, 324].

Even when an effective TCR is deployed, the short persistence of activated T cells will result in reduced therapeutic efficacy, especially in the face of compromised effector function and inhibitory receptor expression of T cells owing to the TME [325]. CD8 T cell dysfunction can be characterized by inability to secrete IL-2, loss of proliferative capacity, and inability to secrete TNF-a and interferon; these functional impairments are incremental and are accompanied by increased expression of inhibitory receptors [326] and decreased numbers of CD4 T cells, which have been shown to inhibit CTL depletion [327].

Regarding the natural exhaustion and escape that occurs in endogenous T cells in the TME, tissue PD-1 upregulation inhibits the function of T cells and induces them toward terminal differentiation. Moreover, modified T cells from TME tend to perform poorly in terms of efficacy [328].

There are a range of optimization options to extend the effective time of TCR-T cells, like removing dysfunctional cells from the circulation to provide opportunities for preferential and stable proliferation of effector and memory T cells. Optimization of the metabolic environment, where hypoxia and mitochondrial dysfunction have been found to be associated with T cell depletion. Glycolytic metabolism, mitochondrial respiration, and metabolites can decisively influence the development and function of T cell populations, including CD8 T cells and regulatory T (Treg) cells [329, 330]. The acidic, hypoxic, nutrient-uptake environment created by the TME in solid malignancies is detrimental to the metabolism of effector T cells. The TME affects key enzymes in glycolysis and respiration, decreasing the glycolytic capacity of infiltrating T cells and leading to their dysfunction. Improved metabolic reprogramming strategies for CD8 T cells could improve the effectiveness ACT [331, 332]. Upregulation of glucose transporters and amino acid transporters in engineered T cells improve the metabolic capacity and subsequent activity of ACT through elevated expression of certain key pro-metabolic factors, such as mTORC1, and cellular proliferation factors, such as c-Myc [333, 334]. Currently, researchers are attempting to improve the uptake rate and metabolic capacity of T cells competing for nutrients and thus their effects through the development of tumor-cell-selective inhibitors of glucose uptake and metabolism in the TME.

In addition, CD8 TILs in hypoglycemia and hypoxia can continue to be active through metabolic pathways that enhance peroxisome activation signaling and fatty acid catabolism, and promotion of fatty acid bypass metabolism has also been shown to enhance the tumor-suppressive ability of TILs [335]. Moreover, in highly glycolytic tumor subtypes, Treg cells were found to promote NFAT-1 translocation and upregulate surface PD-1 expression by actively transporting lactate in a low-glucose and high-lactate TME. Upon PD-1 inhibitor administration, the Treg cells strongly competed with CD8 T cells, enhancing TME inhibition and interfering with therapeutic efficacy [336]. Therefore, predicting glycolytic and lactate modulators of TME is likely to be instrumental in enhancing the adjuvant capacity of ACT activity. Some “preservative” drugs to reprogram the T-cell state are also being investigated, like the use of metformin to regulate CD8 T cell differentiation, which elevated the conversion of T cells to a memory stem-like phenotype, and promoted cytotoxicity in vivo [337, 338].

Incorporation of cytokines has promotion of T cell growth factors, in addition to the benefits derived from lymphocyte pre-elimination, also has the effect of avoiding intrinsic immune cell competition for these cytokines [49]. Improvements have been achieved by antagonizing co-inhibitory signaling molecules such as PD-1 by co-infusion of signal blocking antibodies with engineered T cells [5, 339], or by modification of T cells expressing the PD-1 negative receptor; the anti-tumor efficacy of an IL-6 and PD-1 antibody blockade combination [340] and knockdown of the minus-regulatory protein site using precision editing with CRISPR/Cas9 also enhanced the anti-tumor function of transgenic T cells [341].

#### Carrier cell optimization

The expectation that a uniform allogeneic source of T cells will have advantages for production has led to calls for improved strategies to reduce the risk of GVHD; there has also been optimization of the primitive naïve state and differentiation capacity of the T cells to avoid early emergence of an end-stage T cell depletion state. Studies have also used patient-personalized solid tumor endogenous-derived T cells, such as tumor antigen-specific T cells, which have the potential to produce TCR-T cells [317]. The use of different types of T cell to construct TCR-T cell can maintain the anti-tumor effect and avoid adverse effects [342]. Natural killer (NK) T cells are especially interesting because they are non-allogeneic reactive. Current CAR-T strategies have included the use of NK T cells and other innate-like T lymphocytes “piggybacking” on engineered T cell receptors, an approach that is exempt from the limitations of HLA molecules and has the potential to directly target tumor cells with low-density antigens [343]. NK cells as recipient cells also naturally avoid mismatches triggered by endogenous TCR expression [344]. For surface modification of T cells, functional enhancements and on/off control of T cells have been achieved by introducing the concept of multiple antigen–antibody targeting axes, or multi-gated strategies. Co-stimulatory switch receptors prevent depletion of genetically engineered T cells and may increase their persistence [345–347]. Switch receptors consist of the extracellular portion of inhibitory receptors (e.g., PD-1, TIGIT, TIM-3) and the intracellular signaling domain of co-stimulatory receptors (e.g., CD28, 4-1BB). For example, targeting of low ACT homing in solid tumors, enhanced in vivo homing, and killing of antigen-specific CTLs by cell surface fucosylated CTLs have been demonstrated in a mouse model [348].

## Prospects for combination therapies

### Joint applications

**Fig. 4:**
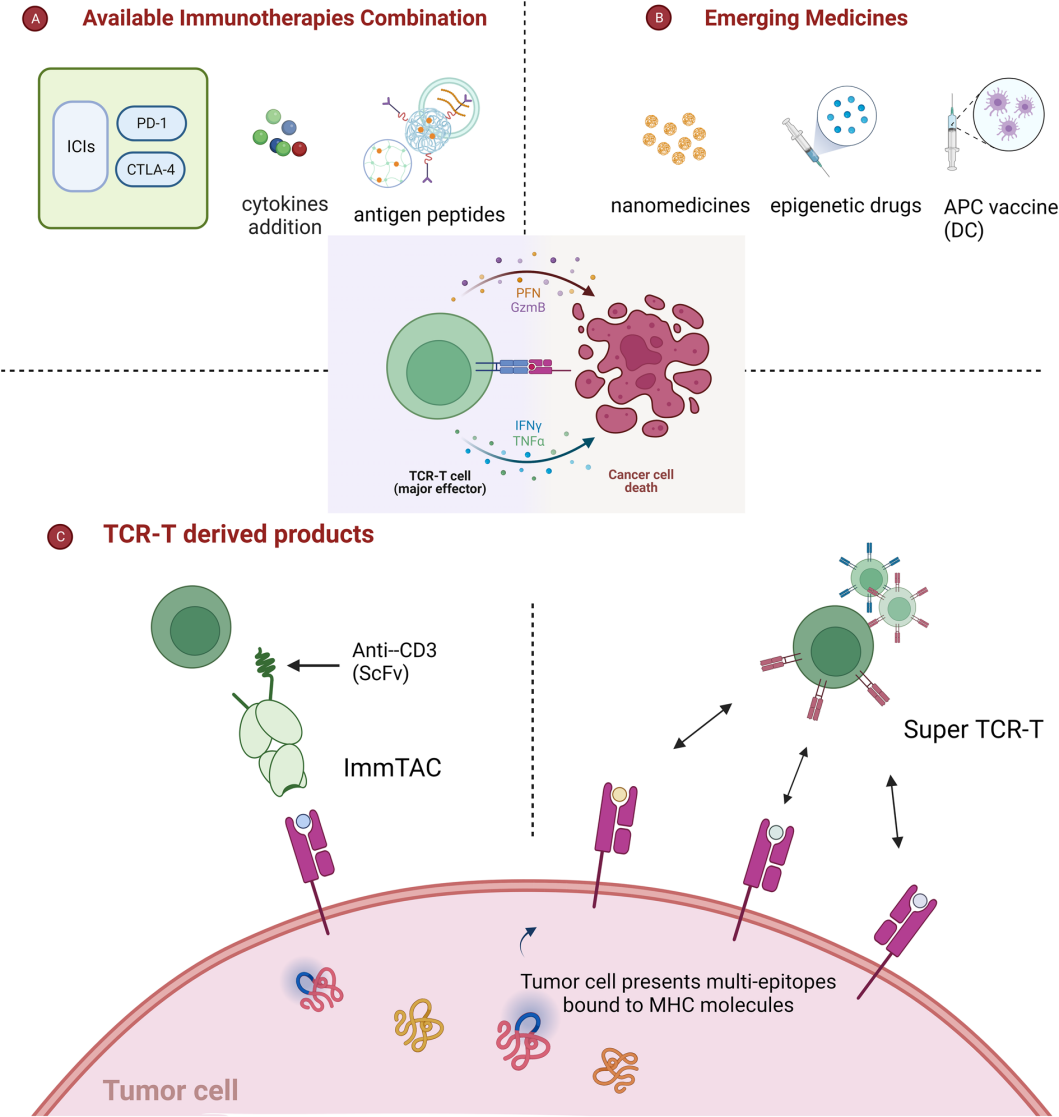
The outlook for TCR-T development. **A** Amplifications of TCR-Ts in conjunction with the main existing immunotherapies to improve efficacy. **B** Amplifications of TCR-Ts in conjunction with some new drugs. **C** Some derivatives of TCR-T: immune-mobilizing monoclonal TCRs Against Cancer (ImmTACs) are not restricted by fixed HLA molecular typing; and Super TCR-T is able to recognize multiple antigenic epitopes of a tumor cell

The body's anti-tumor immune response is complex, multi-component, and not a simple series reaction. TCR-T cell therapies could be combined with other immunotherapies, including ICIs, cytokines, such as IFN-a, monoclonal antibodies targeting specific receptors, and tumor vaccines targeting modifications of tumor cells or APCs (Fig. 4A, B). Recent advances in the direction of anti-tumor nanomedicines, including targeting of ACT cells for prolongation of somatic circulation and inhibition of degradation, have created the possibility of advancing the widespread use of ACT therapy in cancer. Various options for improving the TME are available, and the TME improvements demonstrated in recent studies are applicable to enhancing the efficacy of engineered T cells.

#### ICIs

Although ineffective on their own for certain malignancy outcomes, the commonly used PD-1/PD-L1 inhibitors and anti-CTLA-4 antibodies [349], 350, in combination with ACT, show efficacy in the treatment of various solid malignancies, with each compensating for the deficiencies of the other. In addition, adenosinergic signaling has been found to be an important tumor immunometabolic checkpoint, and adenosine axis blockers have been shown to have promising anti-tumor activity in combination with ACT [351].

#### Anti-tumor cytokines

IL-2, IL-7, IL-15, and IL-21 have been shown to prolong the survival time of memory and naïve T cells in vitro and to favor T cell proliferation [334, 352, 353], but their differentiation-promoting effects on T cells may lead to depletion of subsequent products. IL-18, a member of the IL-1 family, is a pro-inflammatory cytokine capable of promoting a type I immune response and activating a variety of immune cell types, such as stimulating NK cells and promoting the transformation of Th1 cells [354]. The introduction of secreted biologically responsive mature IL18 to enhanced the anti-tumor capacity of Pmel-1-specific T cells infused into melanoma B16F10 hormonal mice to secrete IFN-γ and express CD25, and reduced the aggregation of immune-suppressor cells in the TME, effectively prolonging the survival rate of the mice. In a human melanoma xenograft model, additional transduction of NY-ESO-1-specific TCR-T cells expressing active IL18 resulted in a significant increase in the number of viable T cells in peripheral blood and inhibition of tumor progression [355]. The promotional effect of IL-18 on T cell survival and tumor killing activity was again demonstrated in a recent CAR-T model of advanced refractory solid tumors and in anti-tumor-engineered T cells with increased transformation of the memory phenotype, reduced depletion, and maintenance of a more durable response [356]. Moreover, IL-18 did not cause relevant therapeutic toxicity in patients [357]. Thus, IL-18 should be available as an effective adjuvant anti-cancer factor in combination with TCR-T cell therapies, as it would have multiple benefits. In addition, fusion protein complexes combining IL-12, IL-15, and IL-18 signaling have been developed and validated in an in vivo model, which could promote memory-type differentiation of NK cells and improve their metabolism as well as enhancing anti-tumor cytotoxicity such as secretion of IFN-γ in the short term. Each of them shows a positive promotional effect on TCR-T cells [358], suggesting that such multi-cytokine complexes would be beneficial for TCR-T cell therapies. Given their respective positive promotional effects on TCR-T cells, these multi-cytokine complexes are also likely to have potential for the development and applications of effector enhancement and functional modulation of TCR-T cells.

#### Tumor vaccines

APC vaccines such as DC vaccines have been shown to enhance TCR-T expansion and tumor suppression following TCR-T cell vaccination; in a study that enrolled 14 HLA-A2.1 + patients with metastatic melanoma, signs of tumor regression were observed in 13 of 14 patients following co-vaccination using genetically modified MART-1 TCR-T cells made from autologous T cells and a MART-1 peptide-pulsed DC vaccine. A rapid expansion response of TCR-T cells was observed in vivo, suggesting that dual-cell therapy with concurrent vaccination with DC vaccine further enhances the in vivo expansion of TCR-T cells and exerts anti-tumor effects [36]. By contrast, in a multi-cohort study (n = 6, n = 4) that included 10 patients with advanced sarcoma or melanoma, autologous short-term preparations of NY-ESO-1 TCR-T cells were concomitantly over-transfected with a DC vaccine pulsed with NY-ESO-1 peptide, and signs of tumor regression were observed in two-thirds and one-half of the patients, respectively. However, this study also found that the addition of ipilimumab did not provide any greater clinical benefit [42].

Monocytes from tumor patients with the ability to be APCs were used in a trial to restore APC function using ascites monocyte Toll4 receptor 4 (TLR4) lipopolysaccharide and TLR9 CpG oligodeoxynucleotides. An antibody blocking the IL-10 receptor (IL -10R Ab) restored the function of APCs and could carry and stably conserve a wide range of TAAs, including MUC1, ERBB2, mesothelin, MAGE, PRAME, GPC3, PMEL and TP53. The antibody exhibited potential activation of T cells in vitro and long-term T cell memory effects [359].

Peptide vaccines based on TCR recognition-specific epitopes can also carry information that the target TCR recognizes as homologous immune peptide antigens. Previous studies have demonstrated the ability of such vaccines to reside and remain active in lymph nodes in the region of administration and to enhance the effects of endogenous T cells throughout the body, as well as the ability of the carriers to carry other drugs, such as anti-inflammatory factors [360]. The amphiphile (AMP) vaccine can be conjugated with equipped homologous TCR-T antigen peptides, and results are expected to be published soon. In in vitro assays, pulses of AMP-loaded melanoma antigen-gp100 peptide were co-incubated with mouse DC cells and pre-given homologous TCR-T. This vaccine increased TCR-T cell expression of CD25 and CD69 co-activation markers and secretion of IFN-γ, as well as increasing specific lysis of co-cultured tumor cells [361]. In an in vivo model of homozygous tumor-bearing mice that underwent AMP pre-inoculation of lymph nodes, numbers of TCR-T cells and paracrine immune cells, such as DC cells, in lymph nodes and their ability to secrete IFN-γ increased after TCR-T infusion. Further transcriptomic studies revealed that the AMP-homologous peptide vaccine increased the transcription of genes associated with T cell anti-cancer and immune activation, including the co-stimulatory molecules CD40 and CD86, inflammatory IL12β, IFNγ, and GZMB, as well as TAP1 and TAPBP, which are associated with antigen presentation. The vaccine did not enhance the transcription of immunomodulatory factors associated with T cell depletion or incapacitation, such as FoxP3, CTLA4, and Ceacam1 [361]. In addition, enhanced infiltration of overlying T cells and enhanced expression of reactive substances were found at the tumor site [362]. AMP-matched homologous peptides are easy to fabricate after the design of a TCR-targeting peptide; this series of studies suggests that the combination of an AMP peptide vaccine and relay cell therapy could improve therapeutic efficacy in solid tumors. Furthermore, in a phase 1 clinical trial in a cohort of patients with advanced soft tissue sarcoma, a pullulan nanogel (long peptide antigen) vaccine was used in combination with NY-ESO-1-specific T cells, and one significant tumor shrinkage was observed when all three patients with tumor shrinkage lasting longer than 2 years, none of whom underwent lymphatic clearance. This nanogel vaccine contains a TCR-T cell-recognized NY-ESO-1 epitope, and preclinical studies in an immunosuppressant-resistant mouse model confirmed that the vaccine significantly increased levels of TCR-T cells in draining lymph nodes and tumor tissue [45].

mRNA vaccines can also cooperatively expand specific T cell clones and induce high-intensity T cell response. Besides, they can be sequenced from patient tumour tissues and present personal epitopes. One clinical trial showed the efficacy of the multi-neoantigen autogene cevumeran, individualized neoantigen encoding mRNA lipoplex in the immunotherapy on patients of pancreatic ductal adenocarcinoma. Personalized administration of this vaccine after surgical resection achieved vaccine-induced expansion of neoantigen-specific, long-lived polyfunctional effector T cells and the desired therapeutic effect was observed [363].

#### Epigenetic drugs

Significant upregulation of certain epigenetic factors, such as histone acetylase (HDAC), has been found in various solid tumors, and HDAC inhibitors such as panobinostat show good anti-tumor effects [364]. Aesha et al. found that panobinostat, combined with human T cells transduced with an anti-Her2 CAR and a gp100-TCR, enhanced the transformation of gp100-directed T cells into a central memory phenotype while achieving effective clearance of human pancreatic cancer grafts in a mouse model [365].

#### Parental intermediaries

Addition of membrane amphiphilic markers targeting tumor cells effectively improves engineered T cell recognition and affinity, as a membrane-inserting ligand demonstrated in CAR-T cell trials on solid tumors in vivo [366]. This approach is independent of tumor antigen and tissue of origin and thus has broader applicability in TCR-T, it is based on adding additional common ligands to the heterogeneous population of tumor cells.

### TCR-derived products

Based on engineered TCR-assisted T cell recognition of antigens, TCR or TCR-like therapeutics have been developed that do not require the production of follow-on T cells (Fig. 4C). This avoids the chimeric production and safety issues of genetically engineered modifications of T cells and facilitates production and specification uniformity for such therapies. TCR-mimetic monoclonal antibodies are monoclonal antibodies referencing the structure of the human TCR; they can trigger antibody-, cell-, and complement-mediated cytotoxicity and directly induce apoptosis [367–369]. The main mature products of TCR-derived drugs are immune-mobilizing monoclonal TCRs against cancer (ImmTACs), engineered reagents consisting of soluble specific monoclonal TCRs and anti-CD3 binding domains. ImmTACs are capable of homologous MHC complex recognition by high-affinity TCRs, relocalizing endogenous T cells to kill tumor cells and directing CD3 cross-linking to trigger activation of subsequent T cells. For example, in clinical trials against uveal melanoma, an ImmTAC, tebentafusp (also known as gp100), targeting shared antigens has shown promising results [370, 371].

In addition, the simultaneous application of multiple single-targeted TCR-T cells to counteract tumor antigen escape and infusion of patients with different single-antigen specific CAR-T or TCR-T cell mixtures are viable options. These approaches were recently used in patients with solid tumors, who received up to three new TCR-T cells of different specificities, with no evaluated effect [23]. Notably, multi-targeting of different antigens appears to be a viable strategy to overcome tumor antigen escape, and preliminary results suggest that it may enhance anti-tumor immunity against tumor cells co-expressing multiple antigens. However, this approach is limited by the number of known tumor antigens that can be applied, and the risks of multidrug combinations require consideration. A recent study found that a single TIL extracted from a patient whose advanced solid tumor had regressed after treatment with autologous TILs expressed one individual TCR capable of recognizing all three TAAs. This led to clinical cure for a prolonged period of time [372], indicating hope for the future with respect to the development of a monoclonal TCR-T cell capable of broadly recognizing multiple tumor epitopes. In addition, the ability of TCRs to recognize shared motifs such as x-x-x-A/G-I/L-G-I-x-x-x enables a wider range of effects of TCR-T cell therapies, with the expectation that super "multipronged" TCR-T cells with multiple epitopes targeted by individual engineered T cells will have superior ability to recognize and attack modalities and multiple refractory tumor types [372]. Current studies show promise of continual progress in this regard (e.g., NCT00937625).

## Conclusions

The development of TCR-T cell therapies and the progress made in clinical trials have brought immunotherapy for refractory solid tumors to a new stage. Such therapies are constantly being updated and great advances have been made, including the prediction and screening of tumor neoantigens, assessment of off-target responses, and refinement and establishment of preclinical and clinical trial processes and protocols. Based on existing treatment protocols and clinical trial results, TCR-T cell therapy appears to have unique immunotherapeutic characteristics. Notably, combinations with other therapies, such as radiotherapy and chemotherapy, could improve T cell homing and proliferation, enhance T cell persistence, delay T cell depletion, improve the affinity of tumor peptides, and enhance the active effects. This could improve TCR-T cell oncology treatment and compensate for insufficiencies in previous immunotherapies. By providing more precise and powerful tools for use in the fight against malignant tumors, TCR-T cell therapies promise a brighter future for human tumor immunology.

## Data Availability

Not applicable.

## Abbreviations

ACT: Adoptive cell transfer
APC: Antigen-presenting cell
CAR: Chimeric antigen receptor
CDR: Complementation determining region
CEA: Carcinoembryonic antigen
CGA: Cancer germline antigen
CRS: Cytokine release syndrome
DSB: Double-strand break
FDA: Food and Drug Administration
GVHD: Graft-versus-host disease
HDR: Homology-directed repair
HDRT: HDR template
ICI: Immune checkpoint inhibitor
IL: Interleukin
ImmTAC: Immune-mobilizing monoclonal TCR against cancer
KI: Knock-in
LC: Liquid chromatography
MS: Mass spectrometry
NGS: Next-generation sequencing
NHEJ: Non-homologous end joining
NK: Natural killer
PBL: Peripheral blood lymphocyte
PRAME: Preferentially expressed antigens of melanoma
RECIST: Response evaluation criteria in solid tumors
SB: Sleeping beauty
SNP: Single nucleotide polymorphism
TAA: Tumor-associated antigen
TCR: T cell receptor
TCGA: The Cancer Genome Atlas
TDA: Tissue differentiation antigen
TIL: Tumor-infiltrating lymphocyte
TIR: Terminal inverted repeat
TSA: Tumor-specific antigen
WES: Whole-exome sequencing

## References

[1] Feugier P (2015). A review of rituximab, the first anti-CD20 monoclonal antibody used in the treatment of B non-Hodgkin's lymphomas. Future Oncol.

[2] Adusumilli PS, Zauderer MG, Rivière I, Solomon SB, Rusch VW, O'Cearbhaill RE (2021). A phase I trial of regional mesothelin-targeted CAR T-cell therapy in patients with malignant pleural disease, in combination with the anti-PD-1 agent pembrolizumab. Cancer Discov.

[3] Verdegaal E, Kooij MKVD, Visser M, Minne CVD, Bruin LD, Meij P (2020). Low-dose interferon-alpha preconditioning and adoptive cell therapy in patients with metastatic melanoma refractory to standard (immune) therapies: a phase I/II study. J ImmunoTherapy of Cancer..

[4] Zacharakis N, Chinnasamy H, Black M, Xu H, Lu YC, Zheng Z (2018). Immune recognition of somatic mutations leading to complete durable regression in metastatic breast cancer. Nat Med.

[5] Cherkassky L, Morello A, Villena-Vargas J, Feng Y, Dimitrov DS, Jones DR (2016). Human CAR T cells with cell-intrinsic PD-1 checkpoint blockade resist tumor-mediated inhibition. J Clin Invest.

[6] Paijens ST, Vledder A, de Bruyn M, Nijman HW (2021). Tumor-infiltrating lymphocytes in the immunotherapy era. Cell Mol Immunol.

[7] Zhang P, Zhang G, Wan X (2023). Challenges and new technologies in adoptive cell therapy. J Hematol Oncol.

[8] Zhao L, Cao YJ (2019). Engineered T cell therapy for cancer in the clinic. Front Immunol.

[9] Antonucci L, Canciani G, Mastronuzzi A, Carai A, Del Baldo G, Del Bufalo F (2022). CAR-T therapy for pediatric high-grade gliomas: peculiarities, current investigations and future strategies. Front Immunol.

[10] Holstein SA, Lunning MA (2020). CAR T-cell therapy in hematologic malignancies: a voyage in progress. Clin Pharmacol Ther.

[11] Huang R, Li X, He Y, Zhu W, Gao L, Liu Y (2020). Recent advances in CAR-T cell engineering. J Hematol Oncol.

[12] Ma S, Li X, Wang X, Cheng L, Li Z, Zhang C (2019). Current progress in CAR-T cell therapy for solid tumors. Int J Biol Sci.

[13] Benmebarek MR, Karches CH, Cadilha BL, Lesch S, Endres S, Kobold S (2019). Killing mechanisms of chimeric antigen receptor (CAR) T cells. Int J Mol Sci.

[14] Srivastava S, Furlan SN, Jaeger-Ruckstuhl CA, Sarvothama M, Berger C, Smythe KS (2021). Immunogenic chemotherapy enhances recruitment of CAR-T cells to lung tumors and improves antitumor efficacy when combined with checkpoint blockade. Cancer Cell.

[15] MacKay M, Afshinnekoo E, Rub J, Hassan C, Khunte M, Baskaran N (2020). The therapeutic landscape for cells engineered with chimeric antigen receptors. Nat Biotechnol.

[16] Alcantara M, Du Rusquec P, Romano E (2020). Current clinical evidence and potential solutions to increase benefit of CAR T-cell therapy for patients with solid tumors. Oncoimmunology.

[17] Young RM, Engel NW, Uslu U, Wellhausen N, June CH (2022). Next-generation CAR T-cell therapies. Cancer Discov.

[18] Hong M, Clubb JD, Chen YY (2020). Engineering CAR-T cells for next-generation cancer therapy. Cancer Cell.

[19] Mackensen A, Haanen J, Koenecke C, Alsdorf W, Wagner-Drouet E, Borchmann P (2023). CLDN6-specific CAR-T cells plus amplifying RNA vaccine in relapsed or refractory solid tumors: the phase 1 BNT211-01 trial. Nat Med.

[20] Gastric cancer CAR T-cell target antigen ID'd. Cancer Discov. 2021;11(12):2954. 10.1158/2159-8290.CD-NB2021-0390 34642170

[21] Kim SP, Vale NR, Zacharakis N, Krishna S, Yu Z, Gasmi B (2022). Adoptive cellular therapy with autologous tumor-infiltrating lymphocytes and T-cell receptor-engineered T cells targeting common p53 neoantigens in human solid tumors. Cancer Immunol Res.

[22] Leidner R, Sanjuan Silva N, Huang H, Sprott D, Zheng C, Shih YP (2022). Neoantigen T-cell receptor gene therapy in pancreatic cancer. N Engl J Med.

[23] Foy SP, Jacoby K, Bota DA, Hunter T, Pan Z, Stawiski E (2023). Non-viral precision T cell receptor replacement for personalized cell therapy. Nature.

[24] Kuball J, Dossett ML, Wolfl M, Ho WY, Voss RH, Fowler C (2007). Facilitating matched pairing and expression of TCR chains introduced into human T cells. Blood.

[25] Kondo K, Ohigashi I, Takahama Y (2019). Thymus machinery for T-cell selection. Int Immunol.

[26] Kass I, Buckle AM, Borg NA (2014). Understanding the structural dynamics of TCR-pMHC complex interactions. Trends Immunol.

[27] Harris DT, Kranz DM (2016). Adoptive T cell therapies: a comparison of T cell receptors and chimeric antigen receptors. Trends Pharmacol Sci.

[28] Sun ZJ, Kim KS, Wagner G, Reinherz EL (2001). Mechanisms contributing to T cell receptor signaling and assembly revealed by the solution structure of an ectodomain fragment of the CD3 epsilon gamma heterodimer. Cell.

[29] Bleakley M, Riddell SR (2011). Exploiting T cells specific for human minor histocompatibility antigens for therapy of leukemia. Immunol Cell Biol.

[30] Meij P, Jedema I, van der Hoorn MA, Bongaerts R, Cox L, Wafelman AR (2012). Generation and administration of HA-1-specific T-cell lines for the treatment of patients with relapsed leukemia after allogeneic stem cell transplantation: a pilot study. Haematologica.

[31] Pilunov A, Romaniuk DS, Shmelev A, Sheetikov S, Gabashvili AN, Khmelevskaya A (2023). Transgenic HA-1-specific CD8(+) T-lymphocytes selectively target leukemic cells. Cancers (Basel)..

[32] van Balen P, Jedema I, van Loenen MM, de Boer R, van Egmond HM, Hagedoorn RS (2020). HA-1H T-cell receptor gene transfer to redirect virus-specific T cells for treatment of hematological malignancies after allogeneic stem cell transplantation: a phase 1 clinical study. Front Immunol.

[33] Dossa RG, Cunningham T, Sommermeyer D, Medina-Rodriguez I, Biernacki MA, Foster K (2018). Development of T-cell immunotherapy for hematopoietic stem cell transplantation recipients at risk of leukemia relapse. Blood.

[34] Morgan RA, Dudley ME, Wunderlich JR, Hughes MS, Yang JC, Sherry RM (2006). Cancer regression in patients after transfer of genetically engineered lymphocytes. Science.

[35] Johnson LA, Morgan RA, Dudley ME, Cassard L, Yang JC, Hughes MS (2009). Gene therapy with human and mouse T-cell receptors mediates cancer regression and targets normal tissues expressing cognate antigen. Blood.

[36] Chodon T, Comin-Anduix B, Chmielowski B, Koya RC, Wu Z, Auerbach M (2014). Adoptive transfer of MART-1 T-cell receptor transgenic lymphocytes and dendritic cell vaccination in patients with metastatic melanoma. Clin Cancer Res.

[37] Rohaan MW, Gomez-Eerland R, van den Berg JH, Geukes Foppen MH, van Zon M, Raud B (2022). MART-1 TCR gene-modified peripheral blood T cells for the treatment of metastatic melanoma: a phase I/IIa clinical trial. Immunooncol Technol.

[38] Märkl F, Benmebarek MR, Keyl J, Cadilha BL, Geiger M, Karches C (2023). Bispecific antibodies redirect synthetic agonistic receptor modified T cells against melanoma. J Immunother Cancer.

[39] Hassan R, Butler M, O'Cearbhaill RE, Oh DY, Johnson M, Zikaras K (2023). Mesothelin-targeting T cell receptor fusion construct cell therapy in refractory solid tumors: phase 1/2 trial interim results. Nat Med.

[40] Robbins PF, Morgan RA, Feldman SA, Yang JC, Sherry RM, Dudley ME (2011). Tumor regression in patients with metastatic synovial cell sarcoma and melanoma using genetically engineered lymphocytes reactive with NY-ESO-1. J Clin Oncol.

[41] Robbins PF, Kassim SH, Tran TL, Crystal JS, Morgan RA, Feldman SA (2015). A pilot trial using lymphocytes genetically engineered with an NY-ESO-1-reactive T-cell receptor: long-term follow-up and correlates with response. Clin Cancer Res.

[42] Nowicki TS, Berent-Maoz B, Cheung-Lau G, Huang RR, Wang X, Tsoi J (2019). A pilot trial of the combination of transgenic NY-ESO-1-reactive adoptive cellular therapy with dendritic cell vaccination with or without ipilimumab. Clin Cancer Res.

[43] D'Angelo SP, Melchiori L, Merchant MS, Bernstein D, Glod J, Kaplan R (2018). Antitumor activity associated with prolonged persistence of adoptively transferred NY-ESO-1 (c259)T cells in synovial sarcoma. Cancer Discov.

[44] Ramachandran I, Lowther DE, Dryer-Minnerly R, Wang R, Fayngerts S, Nunez D (2019). Systemic and local immunity following adoptive transfer of NY-ESO-1 SPEAR T cells in synovial sarcoma. J Immunother Cancer.

[45] Ishihara M, Nishida Y, Kitano S, Kawai A, Muraoka D, Momose F (2023). A phase 1 trial of NY-ESO-1-specific TCR-engineered T-cell therapy combined with a lymph node-targeting nanoparticulate peptide vaccine for the treatment of advanced soft tissue sarcoma. Int J Cancer.

[46] Tsuchida CA, Brandes N, Bueno R, Trinidad M, Mazumder T, Yu B (2023). Mitigation of chromosome loss in clinical CRISPR-Cas9-engineered T cells. Cell.

[47] Stadtmauer EA, Faitg TH, Lowther DE, Badros AZ, Chagin K, Dengel K (2019). Long-term safety and activity of NY-ESO-1 SPEAR T cells after autologous stem cell transplant for myeloma. Blood Adv.

[48] Blumenschein GR, Devarakonda S, Johnson M, Moreno V, Gainor J, Edelman MJ (2022). Phase I clinical trial evaluating the safety and efficacy of ADP-A2M10 SPEAR T cells in patients with MAGE-A10(+) advanced non-small cell lung cancer. J Immunother Cancer.

[49] Kageyama S, Ikeda H, Miyahara Y, Imai N, Ishihara M, Saito K (2015). Adoptive transfer of MAGE-A4 T-cell receptor gene-transduced lymphocytes in patients with recurrent esophageal cancer. Clin Cancer Res.

[50] Hong DS, Van Tine BA, Biswas S, McAlpine C, Johnson ML, Olszanski AJ (2023). Autologous T cell therapy for MAGE-A4(+) solid cancers in HLA-A*02(+) patients: a phase 1 trial. Nat Med.

[51] Linette GP, Stadtmauer EA, Maus MV, Rapoport AP, Levine BL, Emery L (2013). Cardiovascular toxicity and titin cross-reactivity of affinity-enhanced T cells in myeloma and melanoma. Blood.

[52] Morgan RA, Chinnasamy N, Abate-Daga D, Gros A, Robbins PF, Zheng Z (2013). Cancer regression and neurological toxicity following anti-MAGE-A3 TCR gene therapy. J Immunother.

[53] Lu YC, Parker LL, Lu T, Zheng Z, Toomey MA, White DE (2017). Treatment of patients with metastatic cancer using a major histocompatibility complex class II-restricted T-cell receptor targeting the cancer germline antigen MAGE-A3. J Clin Oncol.

[54] Boël P, Wildmann C, Sensi ML, Brasseur R, Renauld JC, Coulie P (1995). BAGE: a new gene encoding an antigen recognized on human melanomas by cytolytic T lymphocytes. Immunity.

[55] Ishihara M, Kageyama S, Miyahara Y, Ishikawa T, Ueda S, Soga N (2020). MAGE-A4, NY-ESO-1 and SAGE mRNA expression rates and co-expression relationships in solid tumours. BMC Cancer.

[56] Adams SP, Sahota SS, Mijovic A, Czepulkowski B, Padua RA, Mufti GJ (2002). Frequent expression of HAGE in presentation chronic myeloid leukaemias. Leukemia.

[57] Roman-Gomez J, Jimenez-Velasco A, Agirre X, Castillejo JA, Navarro G, San Jose-Eneriz E (2007). Epigenetic regulation of human cancer/testis antigen gene, HAGE, in chronic myeloid leukemia. Haematologica.

[58] Raskin S, Van Pelt S, Toner K, Balakrishnan PB, Dave H, Bollard CM (2021). Novel TCR-like CAR-T cells targeting an HLA∗0201-restricted SSX2 epitope display strong activity against acute myeloid leukemia. Mol Ther Methods Clin Dev.

[59] Rapoport AP, Stadtmauer EA, Binder-Scholl GK, Goloubeva O, Vogl DT, Lacey SF (2015). NY-ESO-1-specific TCR-engineered T cells mediate sustained antigen-specific antitumor effects in myeloma. Nat Med.

[60] Lim SH, Austin S, Owen-Jones E, Robinson L (1999). Expression of testicular genes in haematological malignancies. Br J Cancer.

[61] Wermke M, Alsdorf W, Araujo D, Chatterjee M, Hilf N, Holderried TA (2023). Abstract PR018: IMA203 TCR-T targeting PRAME demonstrates potent anti-tumor activity in patients with different types of metastatic solid tumors. Mol Cancer Ther.

[62] Lo W, Parkhurst M, Robbins PF, Tran E, Lu YC, Jia L (2019). Immunologic recognition of a shared p53 mutated neoantigen in a patient with metastatic colorectal cancer. Cancer Immunol Res.

[63] Malekzadeh P, Yossef R, Cafri G, Paria BC, Lowery FJ, Jafferji M (2020). Antigen experienced T cells from peripheral blood recognize p53 neoantigens. Clin Cancer Res.

[64] Poole A, Karuppiah V, Hartt A, Haidar JN, Moureau S, Dobrzycki T (2022). Therapeutic high affinity T cell receptor targeting a KRAS(G12D) cancer neoantigen. Nat Commun.

[65] Perumal D, Imai N, Laganà A, Finnigan J, Melnekoff D, Leshchenko VV (2020). Mutation-derived neoantigen-specific T-cell responses in multiple myeloma. Clin Cancer Res.

[66] Cafri G, Yossef R, Pasetto A, Deniger DC, Lu YC, Parkhurst M (2019). Memory T cells targeting oncogenic mutations detected in peripheral blood of epithelial cancer patients. Nat Commun.

[67] Tran E, Robbins PF, Lu YC, Prickett TD, Gartner JJ, Jia L (2016). T-cell transfer therapy targeting mutant KRAS in cancer. N Engl J Med.

[68] Tian W, Zhang W, Wang Y, Jin R, Wang Y, Guo H (2022). Recent advances of IDH1 mutant inhibitor in cancer therapy. Front Pharmacol.

[69] Perner F, Perner C, Ernst T, Heidel FH (2019). Roles of JAK2 in aging, inflammation, hematopoiesis and malignant transformation. Cells.

[70] Veatch JR, Lee SM, Fitzgibbon M, Chow IT, Jesernig B, Schmitt T (2018). Tumor-infiltrating BRAFV600E-specific CD4+ T cells correlated with complete clinical response in melanoma. J Clin Invest.

[71] Leisegang M, Kammertoens T, Uckert W, Blankenstein T (2016). Targeting human melanoma neoantigens by T cell receptor gene therapy. J Clin Invest.

[72] Liu H, Liu K, Dong Z (2021). Targeting CDK12 for cancer therapy: function, mechanism, and drug discovery. Cancer Res.

[73] Schwarz S, Schmitz J, Löffler MW, Ghosh M, Rammensee HG, Olshvang E (2022). T cells of colorectal cancer patients' stimulated by neoantigenic and cryptic peptides better recognize autologous tumor cells. J Immunother Cancer.

[74] Robbins PF, Lu YC, El-Gamil M, Li YF, Gross C, Gartner J (2013). Mining exomic sequencing data to identify mutated antigens recognized by adoptively transferred tumor-reactive T cells. Nat Med.

[75] Karimi Dermani F, Gholamzadeh Khoei S, Afshar S, Amini R (2021). The potential role of nucleophosmin (NPM1) in the development of cancer. J Cell Physiol.

[76] Cimen Bozkus C, Roudko V, Finnigan JP, Mascarenhas J, Hoffman R, Iancu-Rubin C (2019). Immune checkpoint blockade enhances shared neoantigen-induced T-cell immunity directed against mutated calreticulin in myeloproliferative neoplasms. Cancer Discov.

[77] Inderberg EM, Walchli S, Myhre MR, Trachsel S, Almasbak H, Kvalheim G (2017). T cell therapy targeting a public neoantigen in microsatellite instable colon cancer reduces in vivo tumor growth. Oncoimmunology.

[78] Durall RT, Huang J, Wojenski L, Huang Y, Gokhale PC, Leeper BA, et al. The BRD4-NUT fusion alone drives malignant transformation of NUT carcinoma. Cancer Res. 2023;83(23):3846–60. 10.1158/0008-5472.CAN-23-2545 PMC10690098 37819236

[79] Desai AV, Robinson GW, Gauvain K, Basu EM, Macy ME, Maese L (2022). Entrectinib in children and young adults with solid or primary CNS tumors harboring NTRK, ROS1, or ALK aberrations (STARTRK-NG). Neuro Oncol.

[80] Doebele RC, Drilon A, Paz-Ares L, Siena S, Shaw AT, Farago AF (2020). Entrectinib in patients with advanced or metastatic NTRK fusion-positive solid tumours: integrated analysis of three phase 1–2 trials. Lancet Oncol.

[81] Amatu A, Sartore-Bianchi A, Siena S (2016). NTRK gene fusions as novel targets of cancer therapy across multiple tumour types. ESMO Open.

[82] Jonna S, Feldman RA, Swensen J, Gatalica Z, Korn WM, Borghaei H (2019). Detection of NRG1 gene fusions in solid tumors. Clin Cancer Res.

[83] Kim GB, Fritsche J, Bunk S, Mahr A, Unverdorben F, Tosh K (2022). Quantitative immunopeptidomics reveals a tumor stroma-specific target for T cell therapy. Sci Transl Med..

[84] Lozano-Rabella M, Garcia-Garijo A, Palomero J, Yuste-Estevanez A, Erhard F, Farriol-Duran R (2023). Exploring the immunogenicity of noncanonical HLA-I tumor ligands identified through proteogenomics. Clin Cancer Res.

[85] Campillo-Davo D, Flumens D, Lion E (2020). The quest for the best: how TCR affinity, avidity, and functional avidity affect TCR-engineered T-cell antitumor responses. Cells.

[86] Shafer P, Kelly LM, Hoyos V (2022). Cancer therapy with TCR-engineered T cells: current strategies, challenges, and prospects. Front Immunol.

[87] Leko V, Rosenberg SA (2020). Identifying and targeting human tumor antigens for T cell-based immunotherapy of solid tumors. Cancer Cell.

[88] Xie N, Shen G, Gao W, Huang Z, Huang C, Fu L (2023). Neoantigens: promising targets for cancer therapy. Signal Transduct Target Ther.

[89] Moore T, Wagner CR, Scurti GM, Hutchens KA, Godellas C, Clark AL (2018). Clinical and immunologic evaluation of three metastatic melanoma patients treated with autologous melanoma-reactive TCR-transduced T cells. Cancer Immunol Immunother.

[90] Parkhurst MR, Yang JC, Langan RC, Dudley ME, Nathan DA, Feldman SA (2011). T cells targeting carcinoembryonic antigen can mediate regression of metastatic colorectal cancer but induce severe transient colitis. Mol Ther.

[91] Ishihara M, Kitano S, Kageyama S, Miyahara Y, Yamamoto N, Kato H (2022). NY-ESO-1-specific redirected T cells with endogenous TCR knockdown mediate tumor response and cytokine release syndrome. J Immunother Cancer.

[92] Brohl AS, Sindiri S, Wei JS, Milewski D, Chou HC, Song YK (2021). Immuno-transcriptomic profiling of extracranial pediatric solid malignancies. Cell Rep.

[93] Wadelin F, Fulton J, McEwan PA, Spriggs KA, Emsley J, Heery DM (2010). Leucine-rich repeat protein PRAME: expression, potential functions and clinical implications for leukaemia. Mol Cancer.

[94] Doran SL, Stevanović S, Adhikary S, Gartner JJ, Jia L, Kwong MLM (2019). T-cell receptor gene therapy for human papillomavirus-associated epithelial cancers: a first-in-human, phase I/II study. J Clin Oncol.

[95] Nagarsheth NB, Norberg SM, Sinkoe AL, Adhikary S, Meyer TJ, Lack JB (2021). TCR-engineered T cells targeting E7 for patients with metastatic HPV-associated epithelial cancers. Nat Med.

[96] Meng F, Zhao J, Tan AT, Hu W, Wang SY, Jin J (2021). Immunotherapy of HBV-related advanced hepatocellular carcinoma with short-term HBV-specific TCR expressed T cells: results of dose escalation, phase I trial. Hepatol Int.

[97] Veatch J, Paulson K, Asano Y, Martin L, Lee B, Hall ET (2022). Merkel polyoma virus specific T-cell receptor transgenic T-cell therapy in PD-1 inhibitor refractory Merkel cell carcinoma. J Clin Oncol.

[98] Borden ES, Ghafoor S, Buetow KH, LaFleur BJ, Wilson MA, Hastings KT (2022). NeoScore integrates characteristics of the neoantigen:MHC class I interaction and expression to accurately prioritize immunogenic neoantigens. J Immunol.

[99] Barbier AJ, Jiang AY, Zhang P, Wooster R, Anderson DG (2022). The clinical progress of mRNA vaccines and immunotherapies. Nat Biotechnol.

[100] Garcia-Garijo A, Fajardo CA, Gros A (2019). Determinants for neoantigen identification. Front Immunol.

[101] Lang F, Schrors B, Lower M, Tureci O, Sahin U (2022). Identification of neoantigens for individualized therapeutic cancer vaccines. Nat Rev Drug Discov.

[102] Jensen SM, Potts GK, Ready DB, Patterson MJ (2018). Specific MHC-I peptides are induced using PROTACs. Front Immunol.

[103] Wacker M, Bauer J, Wessling L, Dubbelaar M, Nelde A, Rammensee HG (2023). Immunoprecipitation methods impact the peptide repertoire in immunopeptidomics. Front Immunol.

[104] Kobayashi S, Tokita S, Moniwa K, Kitahara K, Iuchi H, Matsuo K (2023). Proteogenomic identification of an immunogenic antigen derived from human endogenous retrovirus in renal cell carcinoma. JCI Insight..

[105] Pandey K, Wang SS, Mifsud NA, Faridi P, Davenport AJ, Webb AI (2023). A combined immunopeptidomics, proteomics, and cell surface proteomics approach to identify immunotherapy targets for diffuse intrinsic pontine glioma. Front Oncol.

[106] Jaeger AM, Stopfer LE, Ahn R, Sanders EA, Sandel DA, Freed-Pastor WA (2022). Deciphering the immunopeptidome in vivo reveals new tumour antigens. Nature.

[107] Tretter C, de Andrade KN, Pecoraro M, Lange S, Seifert P, von Frankenberg C (2023). Proteogenomic analysis reveals RNA as a source for tumor-agnostic neoantigen identification. Nat Commun.

[108] Cuevas MVR, Hardy MP, Larouche JD, Apavaloaei A, Kina E, Vincent K (2023). BamQuery: a proteogenomic tool to explore the immunopeptidome and prioritize actionable tumor antigens. Genome Biol.

[109] Gee MH, Han A, Lofgren SM, Beausang JF, Mendoza JL, Birnbaum ME (2018). Antigen identification for orphan T cell receptors expressed on tumor-infiltrating lymphocytes. Cell.

[110] Kula T, Dezfulian MH, Wang CI, Abdelfattah NS, Hartman ZC, Wucherpfennig KW (2019). T-Scan: a genome-wide method for the systematic discovery of T cell epitopes. Cell.

[111] Danilova L, Anagnostou V, Caushi JX, Sidhom JW, Guo H, Chan HY (2018). The mutation-associated neoantigen functional expansion of specific T cells (MANAFEST) assay: a sensitive platform for monitoring antitumor immunity. Cancer Immunol Res.

[112] Joglekar AV, Leonard MT, Jeppson JD, Swift M, Li G, Wong S (2019). T cell antigen discovery via signaling and antigen-presenting bifunctional receptors. Nat Methods.

[113] Kisielow J, Obermair FJ, Kopf M (2019). Deciphering CD4(+) T cell specificity using novel MHC-TCR chimeric receptors. Nat Immunol.

[114] Li G, Bethune MT, Wong S, Joglekar AV, Leonard MT, Wang JK (2019). T cell antigen discovery via trogocytosis. Nat Methods.

[115] Cattaneo CM, Battaglia T, Urbanus J, Moravec Z, Voogd R, de Groot R (2023). Identification of patient-specific CD4(+) and CD8(+) T cell neoantigens through HLA-unbiased genetic screens. Nat Biotechnol.

[116] Borden ES, Buetow KH, Wilson MA, Hastings KT (2022). Cancer neoantigens: challenges and future directions for prediction, prioritization, and validation. Front Oncol.

[117] Vasaikar SV, Straub P, Wang J, Zhang B (2018). LinkedOmics: analyzing multi-omics data within and across 32 cancer types. Nucleic Acids Res.

[118] Wen B, Zhang B (2023). PepQuery2 democratizes public MS proteomics data for rapid peptide searching. Nat Commun.

[119] Yang KL, Yu F, Teo GC, Li K, Demichev V, Ralser M (2023). MSBooster: improving peptide identification rates using deep learning-based features. Nat Commun.

[120] Abelin JG, Keskin DB, Sarkizova S, Hartigan CR, Zhang W, Sidney J (2017). Mass spectrometry profiling of HLA-associated peptidomes in mono-allelic cells enables more accurate epitope prediction. Immunity.

[121] Calmeiro J, Carrascal M, Gomes C, Falcao A, Cruz MT, Neves BM (2019). Biomaterial-based platforms for in situ dendritic cell programming and their use in antitumor immunotherapy. J Immunother Cancer.

[122] Robinson J, Barker DJ, Georgiou X, Cooper MA, Flicek P, Marsh SGE (2020). IPD-IMGT/HLA database. Nucleic Acids Res.

[123] Okada M, Shimizu K, Fujii SI (2022). Identification of neoantigens in cancer cells as targets for immunotherapy. Int J Mol Sci.

[124] Lundegaard C, Lamberth K, Harndahl M, Buus S, Lund O, Nielsen M (2008). NetMHC-3.0: accurate web accessible predictions of human, mouse and monkey MHC class I affinities for peptides of length 8–11. Nucleic Acids Res.

[125] Reynisson B, Alvarez B, Paul S, Peters B, Nielsen M (2020). NetMHCpan-4.1 and NetMHCIIpan-4.0: improved predictions of MHC antigen presentation by concurrent motif deconvolution and integration of MS MHC eluted ligand data. Nucleic Acids Res.

[126] O'Donnell T, Rubinsteyn A (2020). High-throughput MHC I ligand prediction using MHCflurry. Methods Mol Biol.

[127] O'Donnell TJ, Rubinsteyn A, Laserson U (2020). MHCflurry 2.0: improved pan-allele prediction of MHC class I-presented peptides by incorporating antigen processing. Cell Syst.

[128] Larsen MV, Lelic A, Parsons R, Nielsen M, Hoof I, Lamberth K (2010). Identification of CD8+ T cell epitopes in the West Nile virus polyprotein by reverse-immunology using NetCTL. PLoS ONE.

[129] Stranzl T, Larsen MV, Lundegaard C, Nielsen M (2010). NetCTLpan: pan-specific MHC class I pathway epitope predictions. Immunogenetics.

[130] Tickotsky N, Sagiv T, Prilusky J, Shifrut E, Friedman N (2017). McPAS-TCR: a manually curated catalogue of pathology-associated T cell receptor sequences. Bioinformatics.

[131] Shugay M, Bagaev DV, Zvyagin IV, Vroomans RM, Crawford JC, Dolton G (2018). VDJdb: a curated database of T-cell receptor sequences with known antigen specificity. Nucleic Acids Res.

[132] Balachandran VP, Luksza M, Zhao JN, Makarov V, Moral JA, Remark R (2017). Identification of unique neoantigen qualities in long-term survivors of pancreatic cancer. Nature.

[133] Gerber HP, Sibener LV, Lee LJ, Gee MH (2020). Identification of antigenic targets. Trends Cancer.

[134] Andreatta M, Karosiene E, Rasmussen M, Stryhn A, Buus S, Nielsen M (2015). Accurate pan-specific prediction of peptide-MHC class II binding affinity with improved binding core identification. Immunogenetics.

[135] Wang L, Lan X (2022). Rapid screening of TCR-pMHC interactions by the YAMTAD system. Cell Discov.

[136] Bonte S, De Munter S, Goetgeluk G, Ingels J, Pille M, Billiet L (2020). T-cells with a single tumor antigen-specific T-cell receptor can be generated in vitro from clinically relevant stem cell sources. Oncoimmunology.

[137] Sundararaman S, Karulin AY, Ansari T, BenHamouda N, Gottwein J, Laxmanan S (2015). High reproducibility of ELISPOT counts from nine different laboratories. Cells.

[138] Möbs C, Schmidt T (2016). Research techniques made simple: monitoring of T-cell subsets using the ELISPOT assay. J Invest Dermatol.

[139] Han A, Glanville J, Hansmann L, Davis MM (2014). Linking T-cell receptor sequence to functional phenotype at the single-cell level. Nat Biotechnol.

[140] Lybaert L, Lefever S, Fant B, Smits E, De Geest B, Breckpot K (2023). Challenges in neoantigen-directed therapeutics. Cancer Cell.

[141] Dolton G, Tungatt K, Lloyd A, Bianchi V, Theaker SM, Trimby A (2015). More tricks with tetramers: a practical guide to staining T cells with peptide-MHC multimers. Immunology.

[142] Zhang T, Warden AR, Li Y, Ding X (2020). Progress and applications of mass cytometry in sketching immune landscapes. Clin Transl Med.

[143] Tran E, Ahmadzadeh M, Lu YC, Gros A, Turcotte S, Robbins PF (2015). Immunogenicity of somatic mutations in human gastrointestinal cancers. Science.

[144] Prickett TD, Crystal JS, Cohen CJ, Pasetto A, Parkhurst MR, Gartner JJ (2016). Durable complete response from metastatic melanoma after transfer of autologous T cells recognizing 10 mutated tumor antigens. Cancer Immunol Res.

[145] Ali M, Foldvari Z, Giannakopoulou E, Böschen ML, Strønen E, Yang W (2019). Induction of neoantigen-reactive T cells from healthy donors. Nat Protoc.

[146] Bentzen AK, Marquard AM, Lyngaa R, Saini SK, Ramskov S, Donia M (2016). Large-scale detection of antigen-specific T cells using peptide-MHC-I multimers labeled with DNA barcodes. Nat Biotechnol.

[147] Zhang SQ, Ma KY, Schonnesen AA, Zhang M, He C, Sun E, et al. High-throughput determination of the antigen specificities of T cell receptors in single cells. Nat Biotechnol. 2018;36:1156–9. 10.1038/nbt.4282. 10.1038/nbt.4282 PMC6728224 30418433

[148] Saini SK, Tamhane T, Anjanappa R, Saikia A, Ramskov S, Donia M (2019). Empty peptide-receptive MHC class I molecules for efficient detection of antigen-specific T cells. Sci Immunol..

[149] Lee MN, Meyerson M (2021). Antigen identification for HLA class I- and HLA class II-restricted T cell receptors using cytokine-capturing antigen-presenting cells. Sci Immunol..

[150] Segaliny AI, Li G, Kong L, Ren C, Chen X, Wang JK (2018). Functional TCR T cell screening using single-cell droplet microfluidics. Lab Chip.

[151] Peng S, Zaretsky JM, Ng AHC, Chour W, Bethune MT, Choi J (2019). Sensitive detection and analysis of neoantigen-specific T cell populations from tumors and blood. Cell Rep.

[152] Ng AHC, Peng S, Xu AM, Noh WJ, Guo K, Bethune MT (2019). MATE-Seq: microfluidic antigen-TCR engagement sequencing. Lab Chip.

[153] Schmitt TM, Aggen DH, Ishida-Tsubota K, Ochsenreither S, Kranz DM, Greenberg PD (2017). Generation of higher affinity T cell receptors by antigen-driven differentiation of progenitor T cells in vitro. Nat Biotechnol.

[154] Li LP, Lampert JC, Chen X, Leitao C, Popović J, Müller W (2010). Transgenic mice with a diverse human T cell antigen receptor repertoire. Nat Med.

[155] Obenaus M, Leitão C, Leisegang M, Chen X, Gavvovidis I, van der Bruggen P (2015). Identification of human T-cell receptors with optimal affinity to cancer antigens using antigen-negative humanized mice. Nat Biotechnol.

[156] Macosko EZ, Basu A, Satija R, Nemesh J, Shekhar K, Goldman M (2015). Highly parallel genome-wide expression profiling of individual cells using nanoliter droplets. Cell.

[157] Lowery FJ, Krishna S, Yossef R, Parikh NB, Chatani PD, Zacharakis N (2022). Molecular signatures of antitumor neoantigen-reactive T cells from metastatic human cancers. Science.

[158] Poschke IC, Hassel JC, Rodriguez-Ehrenfried A, Lindner KAM, Heras-Murillo I, Appel LM (2020). The outcome of ex vivo TIL expansion is highly influenced by spatial heterogeneity of the tumor T-cell repertoire and differences in intrinsic in vitro growth capacity between T-cell clones. Clin Cancer Res.

[159] Hanada KI, Zhao C, Gil-Hoyos R, Gartner JJ, Chow-Parmer C, Lowery FJ (2022). A phenotypic signature that identifies neoantigen-reactive T cells in fresh human lung cancers. Cancer Cell.

[160] Arnaud M, Chiffelle J, Genolet R, Navarro Rodrigo B, Perez MAS, Huber F (2022). Sensitive identification of neoantigens and cognate TCRs in human solid tumors. Nat Biotechnol.

[161] Spindler MJ, Nelson AL, Wagner EK, Oppermans N, Bridgeman JS, Heather JM (2020). Massively parallel interrogation and mining of natively paired human TCRalphabeta repertoires. Nat Biotechnol.

[162] Sudmeier LJ, Hoang KB, Nduom EK, Wieland A, Neill SG, Schniederjan MJ (2022). Distinct phenotypic states and spatial distribution of CD8(+) T cell clonotypes in human brain metastases. Cell Rep Med.

[163] Liu S, Iorgulescu JB, Li S, Borji M, Barrera-Lopez IA, Shanmugam V (2022). Spatial maps of T cell receptors and transcriptomes reveal distinct immune niches and interactions in the adaptive immune response. Immunity.

[164] Gao F, Wang K (2015). Ligation-anchored PCR unveils immune repertoire of TCR-beta from whole blood. BMC Biotechnol.

[165] Li B, Li T, Wang B, Dou R, Zhang J, Liu JS (2017). Ultrasensitive detection of TCR hypervariable-region sequences in solid-tissue RNA-seq data. Nat Genet.

[166] Omer A, Peres A, Rodriguez OL, Watson CT, Lees W, Polak P (2022). T cell receptor beta germline variability is revealed by inference from repertoire data. Genome Med.

[167] Li Y, Moysey R, Molloy PE, Vuidepot AL, Mahon T, Baston E (2005). Directed evolution of human T-cell receptors with picomolar affinities by phage display. Nat Biotechnol.

[168] Robbins PF, Li YF, El-Gamil M, Zhao Y, Wargo JA, Zheng Z (2008). Single and dual amino acid substitutions in TCR CDRs can enhance antigen-specific T cell functions. J Immunol.

[169] Chinnasamy N, Wargo JA, Yu Z, Rao M, Frankel TL, Riley JP (2011). A TCR targeting the HLA-A*0201-restricted epitope of MAGE-A3 recognizes multiple epitopes of the MAGE-A antigen superfamily in several types of cancer. J Immunol.

[170] Dash P, Fiore-Gartland AJ, Hertz T, Wang GC, Sharma S, Souquette A (2017). Quantifiable predictive features define epitope-specific T cell receptor repertoires. Nature.

[171] Bagaev DV, Vroomans RMA, Samir J, Stervbo U, Rius C, Dolton G (2020). VDJdb in 2019: database extension, new analysis infrastructure and a T-cell receptor motif compendium. Nucleic Acids Res.

[172] Springer I, Besser H, Tickotsky-Moskovitz N, Dvorkin S, Louzoun Y (2020). Prediction of specific TCR-peptide binding from large dictionaries of TCR-peptide pairs. Front Immunol.

[173] Yang X, Gao M, Chen G, Pierce BG, Lu J, Weng NP (2015). Structural basis for clonal diversity of the public T cell response to a dominant human cytomegalovirus epitope. J Biol Chem.

[174] Chowell D, Krishna S, Becker PD, Cocita C, Shu J, Tan X (2015). TCR contact residue hydrophobicity is a hallmark of immunogenic CD8+ T cell epitopes. Proc Natl Acad Sci U S A.

[175] Malecek K, Zhong S, McGary K, Yu C, Huang K, Johnson LA (2013). Engineering improved T cell receptors using an alanine-scan guided T cell display selection system. J Immunol Methods.

[176] Li N, Yuan J, Tian W, Meng L, Liu Y (2020). T-cell receptor repertoire analysis for the diagnosis and treatment of solid tumor: a methodology and clinical applications. Cancer Commun (Lond).

[177] Antunes DA, Abella JR, Hall-Swan S, Devaurs D, Conev A, Moll M (2020). HLA-Arena: a customizable environment for the structural modeling and analysis of peptide-HLA complexes for cancer immunotherapy. JCO Clin Cancer Inform.

[178] Riley TP, Hellman LM, Gee MH, Mendoza JL, Alonso JA, Foley KC (2018). T cell receptor cross-reactivity expanded by dramatic peptide-MHC adaptability. Nat Chem Biol.

[179] Lu T, Zhang Z, Zhu J, Wang Y, Jiang P, Xiao X (2021). Deep learning-based prediction of the T cell receptor-antigen binding specificity. Nat Mach Intell.

[180] Bradley P (2023). Structure-based prediction of T cell receptor:peptide-MHC interactions. Elife.

[181] Hendrickx R, Stichling N, Koelen J, Kuryk L, Lipiec A, Greber UF (2014). Innate immunity to adenovirus. Hum Gene Ther.

[182] Manfredi F, Cianciotti BC, Potenza A, Tassi E, Noviello M, Biondi A (2020). TCR redirected T cells for cancer treatment: achievements, hurdles, and goals. Front Immunol.

[183] Fraietta JA, Nobles CL, Sammons MA, Lundh S, Carty SA, Reich TJ (2018). Disruption of TET2 promotes the therapeutic efficacy of CD19-targeted T cells. Nature.

[184] Shah NN, Qin H, Yates B, Su L, Shalabi H, Raffeld M (2019). Clonal expansion of CAR T cells harboring lentivector integration in the CBL gene following anti-CD22 CAR T-cell therapy. Blood Adv.

[185] MacLeod DT, Antony J, Martin AJ, Moser RJ, Hekele A, Wetzel KJ (2017). Integration of a CD19 CAR into the TCR alpha chain locus streamlines production of allogeneic gene-edited CAR T cells. Mol Ther.

[186] Nyberg WA, Ark J, To A, Clouden S, Reeder G, Muldoon JJ (2023). An evolved AAV variant enables efficient genetic engineering of murine T cells. Cell.

[187] Izsvak Z, Khare D, Behlke J, Heinemann U, Plasterk RH, Ivics Z (2002). Involvement of a bifunctional, paired-like DNA-binding domain and a transpositional enhancer in Sleeping Beauty transposition. J Biol Chem.

[188] Wang Y, Wang J, Devaraj A, Singh M, Jimenez Orgaz A, Chen JX (2014). Suicidal autointegration of sleeping beauty and piggyBac transposons in eukaryotic cells. PLoS Genet.

[189] Ivics Z, Izsvak Z, Minter A, Hackett PB (1996). Identification of functional domains and evolution of Tc1-like transposable elements. Proc Natl Acad Sci U S A.

[190] Ivics Z, Hackett PB, Plasterk RH, Izsvak Z (1997). Molecular reconstruction of Sleeping Beauty, a Tc1-like transposon from fish, and its transposition in human cells. Cell.

[191] Holstein M, Mesa-Nuñez C, Miskey C, Almarza E, Poletti V, Schmeer M (2018). Efficient non-viral gene delivery into human hematopoietic stem cells by minicircle Sleeping Beauty transposon vectors. Mol Ther.

[192] Gogol-Döring A, Ammar I, Gupta S, Bunse M, Miskey C, Chen W (2016). Genome-wide profiling reveals remarkable parallels between insertion site selection properties of the MLV retrovirus and the piggyBac transposon in primary human CD4(+) T cells. Mol Ther.

[193] Moldt B, Miskey C, Staunstrup NH, Gogol-Doring A, Bak RO, Sharma N (2011). Comparative genomic integration profiling of Sleeping Beauty transposons mobilized with high efficacy from integrase-defective lentiviral vectors in primary human cells. Mol Ther.

[194] Clauss J, Obenaus M, Miskey C, Ivics Z, Izsvák Z, Uckert W (2018). Efficient non-viral T-cell engineering by sleeping beauty minicircles diminishing DNA toxicity and miRNAs silencing the endogenous T-cell receptors. Hum Gene Ther.

[195] Deniger DC, Pasetto A, Tran E, Parkhurst MR, Cohen CJ, Robbins PF (2016). Stable, nonviral expression of mutated tumor neoantigen-specific T-cell receptors using the sleeping beauty transposon/transposase system. Mol Ther.

[196] Peng PD, Cohen CJ, Yang S, Hsu C, Jones S, Zhao Y (2009). Efficient nonviral Sleeping Beauty transposon-based TCR gene transfer to peripheral blood lymphocytes confers antigen-specific antitumor reactivity. Gene Ther.

[197] Tipanee J, Samara-Kuko E, Gevaert T, Chuah MK, VandenDriessche T (2022). Universal allogeneic CAR T cells engineered with Sleeping Beauty transposons and CRISPR-CAS9 for cancer immunotherapy. Mol Ther.

[198] Lock D, Monjezi R, Brandes C, Bates S, Lennartz S, Teppert K (2022). Automated, scaled, transposon-based production of CAR T cells. J Immunother Cancer.

[199] Tsukiyama T, Asano R, Kawaguchi T, Kim N, Yamada M, Minami N (2011). Simple and efficient method for generation of induced pluripotent stem cells using piggyBac transposition of doxycycline-inducible factors and an EOS reporter system. Genes Cells.

[200] Smith RP, Riordan JD, Feddersen CR, Dupuy AJ (2015). A hybrid adenoviral vector system achieves efficient long-term gene expression in the liver via piggyBac transposition. Hum Gene Ther.

[201] Park TS, Han JY (2012). piggyBac transposition into primordial germ cells is an efficient tool for transgenesis in chickens. Proc Natl Acad Sci U S A.

[202] Ding S, Wu X, Li G, Han M, Zhuang Y, Xu T (2005). Efficient transposition of the piggyBac (PB) transposon in mammalian cells and mice. Cell.

[203] Cary LC, Goebel M, Corsaro BG, Wang HG, Rosen E, Fraser MJ (1989). Transposon mutagenesis of baculoviruses: analysis of Trichoplusia ni transposon IFP2 insertions within the FP-locus of nuclear polyhedrosis viruses. Virology.

[204] Wachtl G, Schad E, Huszar K, Palazzo A, Ivics Z, Tantos A, et al. Functional characterization of the N-terminal disordered region of the piggyBac transposase. Int J Mol Sci. 2022;23(18):10317. 10.3390/ijms231810317 PMC9499001 36142241

[205] Mitra R, Fain-Thornton J, Craig NL (2008). piggyBac can bypass DNA synthesis during cut and paste transposition. EMBO J.

[206] Di Matteo M, Belay E, Chuah MK, Vandendriessche T (2012). Recent developments in transposon-mediated gene therapy. Expert Opin Biol Ther.

[207] Morita D, Nishio N, Saito S, Tanaka M, Kawashima N, Okuno Y (2018). Enhanced expression of anti-CD19 chimeric antigen receptor in piggyBac transposon-engineered T cells. Mol Ther Methods Clin Dev.

[208] Nakazawa Y, Saha S, Galvan DL, Huye L, Rollins L, Rooney CM (2013). Evaluation of long-term transgene expression in piggyBac-modified human T lymphocytes. J Immunother.

[209] Helou L, Beauclair L, Dardente H, Piegu B, Tsakou-Ngouafo L, Lecomte T (2021). The piggyBac-derived protein 5 (PGBD5) transposes both the closely and the distantly related piggyBac-like elements Tcr-pble and Ifp2. J Mol Biol.

[210] Yagyu S, Nakazawa Y (2023). piggyBac-transposon-mediated CAR-T cells for the treatment of hematological and solid malignancies. Int J Clin Oncol.

[211] Koga A, Hori H (2001). The Tol2 transposable element of the medaka fish: an active DNA-based element naturally occurring in a vertebrate genome. Genes Genet Syst.

[212] Skipper KA, Andersen PR, Sharma N, Mikkelsen JG (2013). DNA transposon-based gene vehicles—scenes from an evolutionary drive. J Biomed Sci.

[213] Vrljicak P, Tao S, Varshney GK, Quach HN, Joshi A, LaFave MC (2016). Genome-wide analysis of transposon and retroviral insertions reveals preferential integrations in regions of DNA flexibility. G3 (Bethesda)..

[214] Keng VW, Ryan BJ, Wangensteen KJ, Balciunas D, Schmedt C, Ekker SC (2009). Efficient transposition of Tol2 in the mouse germline. Genetics.

[215] Mackey AS, Redd PS, DeLaurier A, Hancock CN. Codon optimized Tol2 transposase results in increased transient expression of a crystallin-GFP transgene in zebrafish. MicroPubl Biol. 2020;(30). 10.17912/micropub.biology.000268 10.17912/micropub.biology.000268 PMC7326334 32626847

[216] Sandoval-Villegas N, Nurieva W, Amberger M, Ivics Z. Contemporary transposon tools: a review and guide through mechanisms and applications of Sleeping Beauty, piggyBac and Tol2 for genome engineering. Int J Mol Sci. 2021;22(10):5084. 10.3390/ijms22105084 PMC8151067 34064900

[217] Okamoto S, Mineno J, Ikeda H, Fujiwara H, Yasukawa M, Shiku H (2009). Improved expression and reactivity of transduced tumor-specific TCRs in human lymphocytes by specific silencing of endogenous TCR. Cancer Res.

[218] Provasi E, Genovese P, Lombardo A, Magnani Z, Liu PQ, Reik A (2012). Editing T cell specificity towards leukemia by zinc finger nucleases and lentiviral gene transfer. Nat Med.

[219] Beane JD, Lee G, Zheng Z, Mendel M, Abate-Daga D, Bharathan M (2015). Clinical scale zinc finger nuclease-mediated gene editing of PD-1 in tumor infiltrating lymphocytes for the treatment of metastatic melanoma. Mol Ther.

[220] Berdien B, Mock U, Atanackovic D, Fehse B (2014). TALEN-mediated editing of endogenous T-cell receptors facilitates efficient reprogramming of T lymphocytes by lentiviral gene transfer. Gene Ther.

[221] Khan SH (2019). Genome-editing technologies: concept, pros, and cons of various genome-editing techniques and bioethical concerns for clinical application. Mol Ther Nucleic Acids.

[222] Ghaffari S, Khalili N, Rezaei N (2021). CRISPR/Cas9 revitalizes adoptive T-cell therapy for cancer immunotherapy. J Exp Clin Cancer Res.

[223] Yi L, Li J (2016). CRISPR-Cas9 therapeutics in cancer: promising strategies and present challenges. Biochim Biophys Acta.

[224] Popp MW, Maquat LE (2016). Leveraging rules of nonsense-mediated mRNA decay for genome engineering and personalized medicine. Cell.

[225] Shy BR, Vykunta VS, Ha A, Talbot A, Roth TL, Nguyen DN (2023). High-yield genome engineering in primary cells using a hybrid ssDNA repair template and small-molecule cocktails. Nat Biotechnol.

[226] Roth TL, Puig-Saus C, Yu R, Shifrut E, Carnevale J, Li PJ (2018). Reprogramming human T cell function and specificity with non-viral genome targeting. Nature.

[227] Shahryari A, Moya N, Siehler J, Wang X, Burtscher I, Lickert H (2021). Increasing gene editing efficiency for CRISPR-Cas9 by small RNAs in pluripotent stem cells. CRISPR J.

[228] Bernard BE, Landmann E, Jeker LT, Schumann K (2022). CRISPR/Cas-based human T cell engineering: basic research and clinical application. Immunol Lett.

[229] Nguyen DN, Roth TL, Li PJ, Chen PA, Apathy R, Mamedov MR (2020). Polymer-stabilized Cas9 nanoparticles and modified repair templates increase genome editing efficiency. Nat Biotechnol.

[230] Janik E, Niemcewicz M, Ceremuga M, Krzowski L, Saluk-Bijak J, Bijak M. Various Aspects of a Gene Editing System-CRISPR-Cas9. Int J Mol Sci. 2020;21(24):9604. 10.3390/ijms21249604 PMC7767219 33339441

[231] Safarzadeh Kozani P, Shokrgozar MA, Evazalipour M, Roudkenar MH (2022). CRISPR/Cas9-medaited knockout of endogenous T-cell receptor in Jurkat cells and generation of NY-ESO-1-specific T cells: an in vitro study. Int Immunopharmacol.

[232] Maganti HB, Kirkham AM, Bailey AJM, Shorr R, Kekre N, Pineault N (2022). Use of CRISPR/Cas9 gene editing to improve chimeric antigen-receptor T cell therapy: a systematic review and meta-analysis of preclinical studies. Cytotherapy.

[233] Forsberg EMV, Lindberg MF, Jespersen H, Alsen S, Bagge RO, Donia M (2019). HER2 CAR-T cells eradicate uveal melanoma and T-cell therapy-resistant human melanoma in IL2 transgenic NOD/SCID IL2 receptor knockout mice. Cancer Res.

[234] Eyquem J, Mansilla-Soto J, Giavridis T, van der Stegen SJ, Hamieh M, Cunanan KM (2017). Targeting a CAR to the TRAC locus with CRISPR/Cas9 enhances tumour rejection. Nature.

[235] Ottaviano G, Georgiadis C, Gkazi SA, Syed F, Zhan H, Etuk A (2022). Phase 1 clinical trial of CRISPR-engineered CAR19 universal T cells for treatment of children with refractory B cell leukemia. Sci Transl Med..

[236] Wang Z, Li N, Feng K, Chen M, Zhang Y, Liu Y (2021). Phase I study of CAR-T cells with PD-1 and TCR disruption in mesothelin-positive solid tumors. Cell Mol Immunol.

[237] Gao Q, Dong X, Xu Q, Zhu L, Wang F, Hou Y (2019). Therapeutic potential of CRISPR/Cas9 gene editing in engineered T-cell therapy. Cancer Med.

[238] Stadtmauer EA, Fraietta JA, Davis MM, Cohen AD, Weber KL, Lancaster E (2020). CRISPR-engineered T cells in patients with refractory cancer. Science.

[239] Stenger D, Stief TA, Kaeuferle T, Willier S, Rataj F, Schober K (2020). Endogenous TCR promotes in vivo persistence of CD19-CAR-T cells compared to a CRISPR/Cas9-mediated TCR knockout CAR. Blood.

[240] Lapteva N, Gilbert M, Diaconu I, Rollins LA, Al-Sabbagh M, Naik S (2019). T-cell receptor stimulation enhances the expansion and function of CD19 chimeric antigen receptor-expressing T cells. Clin Cancer Res.

[241] Hotblack A, Kokalaki EK, Palton MJ, Cheung GW, Williams IP, Manzoor S (2021). Tunable control of CAR T cell activity through tetracycline mediated disruption of protein-protein interaction. Sci Rep.

[242] Giordano-Attianese G, Gainza P, Gray-Gaillard E, Cribioli E, Shui S, Kim S (2020). A computationally designed chimeric antigen receptor provides a small-molecule safety switch for T-cell therapy. Nat Biotechnol.

[243] Stavrou M, Philip B, Traynor-White C, Davis CG, Onuoha S, Cordoba S (2018). A rapamycin-activated caspase 9-based suicide gene. Mol Ther.

[244] Balke-Want H, Keerthi V, Gkitsas N, Mancini AG, Kurgan GL, Fowler C (2023). Homology-independent targeted insertion (HITI) enables guided CAR knock-in and efficient clinical scale CAR-T cell manufacturing. Mol Cancer.

[245] Bravo JPK, Liu MS, Hibshman GN, Dangerfield TL, Jung K, McCool RS (2022). Structural basis for mismatch surveillance by CRISPR-Cas9. Nature.

[246] Vakulskas CA, Dever DP, Rettig GR, Turk R, Jacobi AM, Collingwood MA (2018). A high-fidelity Cas9 mutant delivered as a ribonucleoprotein complex enables efficient gene editing in human hematopoietic stem and progenitor cells. Nat Med.

[247] Gaudelli NM, Komor AC, Rees HA, Packer MS, Badran AH, Bryson DI (2017). Programmable base editing of A*T to G*C in genomic DNA without DNA cleavage. Nature.

[248] Webber BR, Lonetree CL, Kluesner MG, Johnson MJ, Pomeroy EJ, Diers MD (2019). Highly efficient multiplex human T cell engineering without double-strand breaks using Cas9 base editors. Nat Commun.

[249] Kosicki M, Tomberg K, Bradley A (2018). Repair of double-strand breaks induced by CRISPR-Cas9 leads to large deletions and complex rearrangements. Nat Biotechnol.

[250] Zhang S, Shen J, Li D, Cheng Y (2021). Strategies in the delivery of Cas9 ribonucleoprotein for CRISPR/Cas9 genome editing. Theranostics.

[251] Seki A, Rutz S (2018). Optimized RNP transfection for highly efficient CRISPR/Cas9-mediated gene knockout in primary T cells. J Exp Med.

[252] Vazquez-Lombardi R, Jung JS, Schlatter FS, Mei A, Mantuano NR, Bieberich F (2022). High-throughput T cell receptor engineering by functional screening identifies candidates with enhanced potency and specificity. Immunity.

[253] Li G, Iyer B, Prasath VBS, Ni Y, Salomonis N (2021). DeepImmuno: deep learning-empowered prediction and generation of immunogenic peptides for T-cell immunity. Brief Bioinform.

[254] Johnson LA, Heemskerk B, Powell DJ, Cohen CJ, Morgan RA, Dudley ME (2006). Gene transfer of tumor-reactive TCR confers both high avidity and tumor reactivity to nonreactive peripheral blood mononuclear cells and tumor-infiltrating lymphocytes. J Immunol.

[255] Stone JD, Chervin AS, Kranz DM (2009). T-cell receptor binding affinities and kinetics: impact on T-cell activity and specificity. Immunology.

[256] Callender GG, Rosen HR, Roszkowski JJ, Lyons GE, Li M, Moore T (2006). Identification of a hepatitis C virus-reactive T cell receptor that does not require CD8 for target cell recognition. Hepatology.

[257] Rees W, Bender J, Teague TK, Kedl RM, Crawford F, Marrack P (1999). An inverse relationship between T cell receptor affinity and antigen dose during CD4(+) T cell responses in vivo and in vitro. Proc Natl Acad Sci U S A.

[258] Ch'ng ACW, Lam P, Alassiri M, Lim TS (2022). Application of phage display for T-cell receptor discovery. Biotechnol Adv.

[259] Loset GA, Berntzen G, Frigstad T, Pollmann S, Gunnarsen KS, Sandlie I (2014). Phage display engineered T cell receptors as tools for the study of tumor peptide-MHC interactions. Front Oncol.

[260] Fukuda N, Ishii J, Shibasaki S, Ueda M, Fukuda H, Kondo A (2007). High-efficiency recovery of target cells using improved yeast display system for detection of protein-protein interactions. Appl Microbiol Biotechnol.

[261] Zhao Y, Bennett AD, Zheng Z, Wang QJ, Robbins PF, Yu LY (2007). High-affinity TCRs generated by phage display provide CD4+ T cells with the ability to recognize and kill tumor cell lines. J Immunol.

[262] Spear TT, Evavold BD, Baker BM, Nishimura MI (2019). Understanding TCR affinity, antigen specificity, and cross-reactivity to improve TCR gene-modified T cells for cancer immunotherapy. Cancer Immunol Immunother.

[263] Liu B, Kolawole EM, Evavold BD (2021). Mechanobiology of T cell activation: to catch a bond. Annu Rev Cell Dev Biol.

[264] Sibener LV, Fernandes RA, Kolawole EM, Carbone CB, Liu F, McAffee D (2018). Isolation of a structural mechanism for uncoupling T cell receptor signaling from peptide-MHC binding. Cell.

[265] Liu Y, Blanchfield L, Ma VP, Andargachew R, Galior K, Liu Z (2016). DNA-based nanoparticle tension sensors reveal that T-cell receptors transmit defined pN forces to their antigens for enhanced fidelity. Proc Natl Acad Sci U S A.

[266] Liu B, Chen W, Evavold BD, Zhu C (2014). Accumulation of dynamic catch bonds between TCR and agonist peptide-MHC triggers T cell signaling. Cell.

[267] Wu P, Zhang T, Liu B, Fei P, Cui L, Qin R (2019). Mechano-regulation of peptide-MHC Class I conformations determines TCR antigen recognition. Mol Cell.

[268] Zhao X, Kolawole EM, Chan W, Feng Y, Yang X, Gee MH (2022). Tuning T cell receptor sensitivity through catch bond engineering. Science.

[269] Wang JH (2020). T cell receptors, mechanosensors, catch bonds and immunotherapy. Prog Biophys Mol Biol.

[270] Pettmann J, Awada L, Rozycki B, Huhn A, Faour S, Kutuzov M (2023). Mechanical forces impair antigen discrimination by reducing differences in T-cell receptor/peptide-MHC off-rates. EMBO J.

[271] Szeto C, Zareie P, Wirasinha RC, Zhang JB, Nguyen AT, Riboldi-Tunnicliffe A (2022). Covalent TCR-peptide-MHC interactions induce T cell activation and redirect T cell fate in the thymus. Nat Commun.

[272] Hellmeier J, Platzer R, Huppa JB, Sevcsik E (2023). A DNA origami-based biointerface to interrogate the spatial requirements for sensitized T-cell antigen recognition. Methods Mol Biol.

[273] Cameron BJ, Gerry AB, Dukes J, Harper JV, Kannan V, Bianchi FC (2013). Identification of a Titin-derived HLA-A1-presented peptide as a cross-reactive target for engineered MAGE A3-directed T cells. Sci Transl Med..

[274] Sanderson JP, Crowley DJ, Wiedermann GE, Quinn LL, Crossland KL, Tunbridge HM (2020). Preclinical evaluation of an affinity-enhanced MAGE-A4-specific T-cell receptor for adoptive T-cell therapy. Oncoimmunology.

[275] Luo X, Cui H, Cai L, Zhu W, Yang WC, Patrick M (2020). Selection of a clinical lead TCR targeting alpha-fetoprotein-positive liver cancer based on a balance of risk and benefit. Front Immunol.

[276] Birnbaum ME, Mendoza JL, Sethi DK, Dong S, Glanville J, Dobbins J (2014). Deconstructing the peptide-MHC specificity of T cell recognition. Cell.

[277] Bijen HM, van der Steen DM, Hagedoorn RS, Wouters AK, Wooldridge L, Falkenburg JHF (2018). Preclinical strategies to identify off-target toxicity of high-affinity TCRs. Mol Ther.

[278] Ishii K, Davies JS, Sinkoe AL, Nguyen KA, Norberg SM, McIntosh CP (2023). Multi-tiered approach to detect autoimmune cross-reactivity of therapeutic T cell receptors. Sci Adv.

[279] Karapetyan AR, Chaipan C, Winkelbach K, Wimberger S, Jeong JS, Joshi B (2019). TCR fingerprinting and off-target peptide identification. Front Immunol.

[280] Bentzen AK, Such L, Jensen KK, Marquard AM, Jessen LE, Miller NJ (2018). T cell receptor fingerprinting enables in-depth characterization of the interactions governing recognition of peptide-MHC complexes. Nat Biotechnol.

[281] Fonseca AF, Antunes DA (2023). CrossDome: an interactive R package to predict cross-reactivity risk using immunopeptidomics databases. Front Immunol.

[282] Sun Y, Li F, Sonnemann H, Jackson KR, Talukder AH, Katailiha AS (2021). Evolution of CD8(+) T cell receptor (TCR) engineered therapies for the treatment of cancer. Cells.

[283] Huang W, Percie du Sert N, Vollert J, Rice ASC (2020). General principles of preclinical study design. Handb Exp Pharmacol.

[284] Susukida T, Aoki S, Shirayanagi T, Yamada Y, Kuwahara S, Ito K (2020). HLA transgenic mice: application in reproducing idiosyncratic drug toxicity. Drug Metab Rev.

[285] Donnadieu E, Luu M, Alb M, Anliker B, Arcangeli S, Bonini C (2022). Time to evolve: predicting engineered T cell-associated toxicity with next-generation models. J Immunother Cancer.

[286] Pan Q, Weng D, Liu J, Han Z, Ou Y, Xu B (2023). Phase 1 clinical trial to assess safety and efficacy of NY-ESO-1-specific TCR T cells in HLA-A∗02:01 patients with advanced soft tissue sarcoma. Cell Rep Med.

[287] Tsimberidou AM, Guenther K, Andersson BS, Mendrzyk R, Alpert A, Wagner C (2023). Feasibility and safety of personalized, multi-target, adoptive cell therapy (IMA101): first-in-human clinical trial in patients with advanced metastatic cancer. Cancer Immunol Res.

[288] Hirayama AV, Turtle CJ (2019). Toxicities of CD19 CAR-T cell immunotherapy. Am J Hematol.

[289] Eisenhauer EA, Therasse P, Bogaerts J, Schwartz LH, Sargent D, Ford R (2009). New response evaluation criteria in solid tumours: revised RECIST guideline (version 1.1). Eur J Cancer.

[290] Bohnsack O, Hoos A, Ludajic K (2014). Adaptation and modification of the immune related response criteria (IRRC): IrRECIS. J Clin Oncol.

[291] Seymour L, Bogaerts J, Perrone A, Ford R, Schwartz LH, Mandrekar S (2017). iRECIST: guidelines for response criteria for use in trials testing immunotherapeutics. Lancet Oncol.

[292] van Loenen MM, de Boer R, Amir AL, Hagedoorn RS, Volbeda GL, Willemze R (2010). Mixed T cell receptor dimers harbor potentially harmful neoreactivity. Proc Natl Acad Sci U S A.

[293] Ferrara J, Reddy P, Paczesny S (2010). Immunotherapy through T-cell receptor gene transfer induces severe graft-versus-host disease. Immunotherapy.

[294] Bendle GM, Linnemann C, Hooijkaas AI, Bies L, de Witte MA, Jorritsma A (2010). Lethal graft-versus-host disease in mouse models of T cell receptor gene therapy. Nat Med.

[295] Okada S, Muraoka D, Yasui K, Tawara I, Kawamura A, Okamoto S (2023). T cell receptor gene-modified allogeneic T cells with siRNA for endogenous T cell receptor induce efficient tumor regression without graft-versus-host disease. Cancer Sci.

[296] Rosenberg SA (2010). Of mice, not men: no evidence for graft-versus-host disease in humans receiving T-cell receptor-transduced autologous T cells. Mol Ther.

[297] Turtle CJ, Hanafi LA, Berger C, Gooley TA, Cherian S, Hudecek M (2016). CD19 CAR-T cells of defined CD4+:CD8+ composition in adult B cell ALL patients. J Clin Invest.

[298] Cohen CJ, Li YF, El-Gamil M, Robbins PF, Rosenberg SA, Morgan RA (2007). Enhanced antitumor activity of T cells engineered to express T-cell receptors with a second disulfide bond. Cancer Res.

[299] Haga-Friedman A, Horovitz-Fried M, Cohen CJ (2012). Incorporation of transmembrane hydrophobic mutations in the TCR enhance its surface expression and T cell functional avidity. J Immunol.

[300] Zhang Z, Liu C, Wang M, Sun R, Yang Z, Hua Z (2023). Treating solid tumors with TCR-based chimeric antigen receptor targeting extra domain B-containing fibronectin. J Immunother Cancer.

[301] Bethune MT, Gee MH, Bunse M, Lee MS, Gschweng EH, Pagadala MS (2016). Domain-swapped T cell receptors improve the safety of TCR gene therapy. Elife.

[302] Tao C, Shao H, Zhang W, Bo H, Wu F, Shen H (2017). γδTCR immunoglobulin constant region domain exchange in human αβTCRs improves TCR pairing without altering TCR gene-modified T cell function. Mol Med Rep.

[303] Heather JM, Spindler MJ, Alonso MH, Shui YI, Millar DG, Johnson DS (2022). Stitchr: stitching coding TCR nucleotide sequences from V/J/CDR3 information. Nucleic Acids Res.

[304] Bunse M, Bendle GM, Linnemann C, Bies L, Schulz S, Schumacher TN (2014). RNAi-mediated TCR knockdown prevents autoimmunity in mice caused by mixed TCR dimers following TCR gene transfer. Mol Ther.

[305] Neelapu SS, Tummala S, Kebriaei P, Wierda W, Gutierrez C, Locke FL (2018). Chimeric antigen receptor T-cell therapy—assessment and management of toxicities. Nat Rev Clin Oncol.

[306] Kim ST, Tayar J, Fu S, Ke D, Norry E, Sun A (2021). Newly developed pseudogout arthritis after therapy with MAGE-A4 directed TCR T cells responded to treatment with tocilizumab. J Immunother Cancer.

[307] Gong N, Han X, Xue L, El-Mayta R, Metzloff AE, Billingsley MM (2023). In situ PEGylation of CAR T cells alleviates cytokine release syndrome and neurotoxicity. Nat Mater.

[308] Gejman RS, Chang AY, Jones HF, DiKun K, Hakimi AA, Schietinger A (2018). Rejection of immunogenic tumor clones is limited by clonal fraction. Elife.

[309] McGranahan N, Furness AJ, Rosenthal R, Ramskov S, Lyngaa R, Saini SK (2016). Clonal neoantigens elicit T cell immunoreactivity and sensitivity to immune checkpoint blockade. Science.

[310] Li Y, Hong YK, Wang X, Pandit H, Zheng Q, Yu Y (2022). Epigenetic modulation enhances immunotherapy for pancreatic ductal adenocarcinoma. Clin Transl Immunol.

[311] Rosenthal R, Cadieux EL, Salgado R, Bakir MA, Moore DA, Hiley CT (2019). Neoantigen-directed immune escape in lung cancer evolution. Nature.

[312] Flavahan WA, Gaskell E, Bernstein BE (2017). Epigenetic plasticity and the hallmarks of cancer. Science.

[313] Gardner A, Ruffell B (2016). Dendritic cells and cancer immunity. Trends Immunol.

[314] Crespo J, Sun H, Welling TH, Tian Z, Zou W (2013). T cell anergy, exhaustion, senescence, and stemness in the tumor microenvironment. Curr Opin Immunol.

[315] Lerner EC, Woroniecka KI, D’Anniballe VM, Wilkinson DS, Mohan AA, Lorrey SJ (2023). CD8(+) T cells maintain killing of MHC-I-negative tumor cells through the NKG2D-NKG2DL axis. Nat Cancer..

[316] Thomas S, Mohammed F, Reijmers RM, Woolston A, Stauss T, Kennedy A (2019). Framework engineering to produce dominant T cell receptors with enhanced antigen-specific function. Nat Commun.

[317] He J, Xiong X, Yang H, Li D, Liu X, Li S (2022). Defined tumor antigen-specific T cells potentiate personalized TCR-T cell therapy and prediction of immunotherapy response. Cell Res.

[318] Yee C (2018). Adoptive T cell therapy: points to consider. Curr Opin Immunol.

[319] Wherry EJ, Kurachi M (2015). Molecular and cellular insights into T cell exhaustion. Nat Rev Immunol.

[320] Blackburn SD, Shin H, Haining WN, Zou T, Workman CJ, Polley A (2009). Coregulation of CD8+ T cell exhaustion by multiple inhibitory receptors during chronic viral infection. Nat Immunol.

[321] Chow A, Perica K, Klebanoff CA, Wolchok JD (2022). Clinical implications of T cell exhaustion for cancer immunotherapy. Nat Rev Clin Oncol.

[322] Wang JC, Xu Y, Huang ZM, Lu XJ (2018). T cell exhaustion in cancer: mechanisms and clinical implications. J Cell Biochem.

[323] Eil R, Vodnala SK, Clever D, Klebanoff CA, Sukumar M, Pan JH (2016). Ionic immune suppression within the tumour microenvironment limits T cell effector function. Nature.

[324] Palmer DC, Guittard GC, Franco Z, Crompton JG, Eil RL, Patel SJ (2015). Cish actively silences TCR signaling in CD8+ T cells to maintain tumor tolerance. J Exp Med.

[325] Wherry EJ (2011). T cell exhaustion. Nat Immunol.

[326] Amezquita RA, Kaech SM (2017). Immunology: the chronicles of T-cell exhaustion. Nature.

[327] Lu YJ, Barreira-Silva P, Boyce S, Powers J, Cavallo K, Behar SM (2021). CD4 T cell help prevents CD8 T cell exhaustion and promotes control of Mycobacterium tuberculosis infection. Cell Rep.

[328] Fan M, Li M, Gao L, Geng S, Wang J, Wang Y (2017). Chimeric antigen receptors for adoptive T cell therapy in acute myeloid leukemia. J Hematol Oncol.

[329] Sukumar M, Liu J, Ji Y, Subramanian M, Crompton JG, Yu Z (2013). Inhibiting glycolytic metabolism enhances CD8+ T cell memory and antitumor function. J Clin Invest.

[330] van der Windt GJ, Everts B, Chang CH, Curtis JD, Freitas TC, Amiel E (2012). Mitochondrial respiratory capacity is a critical regulator of CD8+ T cell memory development. Immunity.

[331] Gemta LF, Siska PJ, Nelson ME, Gao X, Liu X, Locasale JW, et al. Impaired enolase 1 glycolytic activity restrains effector functions of tumor-infiltrating CD8(+) T cells. Sci Immunol. 2019;4(31):eaap9520. 10.1126/sciimmunol.aap9520 PMC6824424 30683669

[332] Hay N (2016). Reprogramming glucose metabolism in cancer: can it be exploited for cancer therapy?. Nat Rev Cancer.

[333] Nachef M, Ali AK, Almutairi SM, Lee SH (2021). Targeting SLC1A5 and SLC3A2/SLC7A5 as a potential strategy to strengthen anti-tumor immunity in the tumor microenvironment. Front Immunol.

[334] Alizadeh D, Wong RA, Yang X, Wang D, Pecoraro JR, Kuo CF (2019). IL15 enhances CAR-T cell antitumor activity by reducing mTORC1 activity and preserving their stem cell memory phenotype. Cancer Immunol Res.

[335] Zhang Y, Kurupati R, Liu L, Zhou XY, Zhang G, Hudaihed A (2017). Enhancing CD8(+) T cell fatty acid catabolism within a metabolically challenging tumor microenvironment increases the efficacy of melanoma immunotherapy. Cancer Cell.

[336] Kumagai S, Koyama S, Itahashi K, Tanegashima T, Lin YT, Togashi Y (2022). Lactic acid promotes PD-1 expression in regulatory T cells in highly glycolytic tumor microenvironments. Cancer Cell.

[337] Zhang Z, Li F, Tian Y, Cao L, Gao Q, Zhang C (2020). Metformin enhances the antitumor activity of CD8(+) T lymphocytes via the AMPK-miR-107-Eomes-PD-1 pathway. J Immunol.

[338] Eikawa S, Nishida M, Mizukami S, Yamazaki C, Nakayama E, Udono H (2015). Immune-mediated antitumor effect by type 2 diabetes drug, metformin. Proc Natl Acad Sci U S A.

[339] Chong EA, Melenhorst JJ, Lacey SF, Ambrose DE, Gonzalez V, Levine BL (2017). PD-1 blockade modulates chimeric antigen receptor (CAR)-modified T cells: refueling the CAR. Blood.

[340] Mace TA, Shakya R, Pitarresi JR, Swanson B, McQuinn CW, Loftus S (2018). IL-6 and PD-L1 antibody blockade combination therapy reduces tumour progression in murine models of pancreatic cancer. Gut.

[341] Ren J, Liu X, Fang C, Jiang S, June CH, Zhao Y (2017). Multiplex genome editing to generate universal CAR T cells resistant to PD1 inhibition. Clin Cancer Res.

[342] Zhang Y, Liu Z, Wei W, Li Y (2022). TCR engineered T cells for solid tumor immunotherapy. Exp Hematol Oncol.

[343] Courtney AN, Tian G, Metelitsa LS (2023). Natural killer T cells and other innate-like T lymphocytes as emerging platforms for allogeneic cancer cell therapy. Blood.

[344] Parlar A, Sayitoglu EC, Ozkazanc D, Georgoudaki AM, Pamukcu C, Aras M (2019). Engineering antigen-specific NK cell lines against the melanoma-associated antigen tyrosinase via TCR gene transfer. Eur J Immunol.

[345] Zhao S, Wang C, Lu P, Lou Y, Liu H, Wang T (2021). Switch receptor T3/28 improves long-term persistence and antitumor efficacy of CAR-T cells. J Immunother Cancer.

[346] Hoogi S, Eisenberg V, Mayer S, Shamul A, Barliya T, Cohen CJ (2019). A TIGIT-based chimeric co-stimulatory switch receptor improves T-cell anti-tumor function. J Immunother Cancer.

[347] Liu X, Ranganathan R, Jiang S, Fang C, Sun J, Kim S (2016). A chimeric switch-receptor targeting PD1 augments the efficacy of second-generation CAR T cells in advanced solid tumors. Cancer Res.

[348] Alatrash G, Qiao N, Zhang M, Zope M, Perakis AA, Sukhumalchandra P (2019). Fucosylation enhances the efficacy of adoptively transferred antigen-specific cytotoxic T lymphocytes. Clin Cancer Res.

[349] Puig-Saus C, Sennino B, Peng S, Wang CL, Pan Z, Yuen B (2023). Neoantigen-targeted CD8(+) T cell responses with PD-1 blockade therapy. Nature.

[350] Kabacaoglu D, Ciecielski KJ, Ruess DA, Algül H (2018). Immune checkpoint inhibition for pancreatic ductal adenocarcinoma: current limitations and future options. Front Immunol.

[351] Vigano S, Alatzoglou D, Irving M, Ménétrier-Caux C, Caux C, Romero P (2019). Targeting adenosine in cancer immunotherapy to enhance T-cell function. Front Immunol.

[352] Battram AM, Bachiller M, Lopez V, Fernandez de Larrea C, Urbano-Ispizua A, Martin-Antonio B (2021). IL-15 enhances the persistence and function of BCMA-targeting CAR-T cells compared to IL-2 or IL-15/IL-7 by limiting CAR-T cell dysfunction and differentiation. Cancers (Basel)..

[353] Loschinski R, Bottcher M, Stoll A, Bruns H, Mackensen A, Mougiakakos D (2018). IL-21 modulates memory and exhaustion phenotype of T-cells in a fatty acid oxidation-dependent manner. Oncotarget.

[354] Yasuda K, Nakanishi K, Tsutsui H (2019). Interleukin-18 in health and disease. Int J Mol Sci.

[355] Drakes DJ, Rafiq S, Purdon TJ, Lopez AV, Chandran SS, Klebanoff CA (2020). Optimization of T-cell receptor-modified T cells for cancer therapy. Cancer Immunol Res.

[356] Jaspers JE, Khan JF, Godfrey WD, Lopez AV, Ciampricotti M, Rudin CM (2023). IL-18-secreting CAR T cells targeting DLL3 are highly effective in small cell lung cancer models. J Clin Invest.

[357] Tarhini AA, Millward M, Mainwaring P, Kefford R, Logan T, Pavlick A (2009). A phase 2, randomized study of SB-485232, rhIL-18, in patients with previously untreated metastatic melanoma. Cancer.

[358] Becker-Hapak MK, Shrestha N, McClain E, Dee MJ, Chaturvedi P, Leclerc GM (2021). A fusion protein complex that combines IL-12, IL-15, and IL-18 signaling to induce memory-like NK cells for cancer immunotherapy. Cancer Immunol Res.

[359] Adams SF, Grimm AJ, Chiang CL, Mookerjee A, Flies D, Jean S (2020). Rapid tumor vaccine using Toll-like receptor-activated ovarian cancer ascites monocytes. J Immunother Cancer.

[360] Du G, Sun X. 19—Lymph node targeting for improved potency of cancer vaccine. In: Park K, editor. Biomaterials for cancer therapeutics (Second Edition): Woodhead Publishing; 2020. p. 527–48.

[361] Liu H, Moynihan KD, Zheng Y, Szeto GL, Li AV, Huang B (2014). Structure-based programming of lymph-node targeting in molecular vaccines. Nature.

[362] Drakes DJ, Abbas AM, Shields J, Steinbuck MP, Jakubowski A, Seenappa LM, Haqq CM, DeMuth PC. Lymph Node-Targeted Vaccine Boosting of TCR T-cell Therapy Enhances Antitumor Function and Eradicates Solid Tumors. Cancer Immunol Res. 2024;12(2):214–31. 10.1158/2326-6066.CIR-22-0978 PMC10835214 38270373

[363] Rojas LA, Sethna Z, Soares KC, Olcese C, Pang N, Patterson E (2023). Personalized RNA neoantigen vaccines stimulate T cells in pancreatic cancer. Nature.

[364] Biersack B, Polat S, Höpfner M (2022). Anticancer properties of chimeric HDAC and kinase inhibitors. Semin Cancer Biol.

[365] Ali AI, Wang M, von Scheidt B, Dominguez PM, Harrison AJ, Tantalo DGM (2021). A histone deacetylase inhibitor, panobinostat, enhances chimeric antigen receptor T-cell antitumor effect against pancreatic cancer. Clin Cancer Res.

[366] Zhang AQ, Hostetler A, Chen LE, Mukkamala V, Abraham W, Padilla LT (2023). Universal redirection of CAR T cells against solid tumours via membrane-inserted ligands for the CAR. Nat Biomed Eng.

[367] Liu X, Xu Y, Xiong W, Yin B, Huang Y, Chu J (2022). Development of a TCR-like antibody and chimeric antigen receptor against NY-ESO-1/HLA-A2 for cancer immunotherapy. J Immunother Cancer.

[368] Gerber HP, Presta LG (2022). TCR mimic compounds for pHLA targeting with high potency modalities in oncology. Front Oncol.

[369] Verma B, Jain R, Caseltine S, Rennels A, Bhattacharya R, Markiewski MM (2011). TCR mimic monoclonal antibodies induce apoptosis of tumor cells via immune effector-independent mechanisms. J Immunol.

[370] Dhillon S (2022). Tebentafusp: first approval. Drugs.

[371] Algazi AP, Tsai KK, Shoushtari AN, Munhoz RR, Eroglu Z, Piulats JM (2016). Clinical outcomes in metastatic uveal melanoma treated with PD-1 and PD-L1 antibodies. Cancer.

[372] Dolton G, Rius C, Wall A, Szomolay B, Bianchi V, Galloway SAE (2023). Targeting of multiple tumor-associated antigens by individual T cell receptors during successful cancer immunotherapy. Cell.

